# A quantitative risk assessment framework for mortality due to macroplastic ingestion in seabirds, marine mammals, and sea turtles

**DOI:** 10.1073/pnas.2415492122

**Published:** 2025-11-17

**Authors:** Erin L. Murphy, Britta R. Baechler, Lauren Roman, George H. Leonard, Nicholas J. Mallos, Robson G. Santos, Chelsea M. Rochman

**Affiliations:** ^a^Ocean Conservancy, Washington, DC 20036; ^b^Department of Ecology and Evolutionary Biology, University of Toronto, Toronto, ON M5R 0A3, Canada; ^c^Institute for Marine and Antarctic Studies, University of Tasmania, Hobart, TAS 7005, Australia; ^d^Commonwealth Scientific and Industrial Research Organisation (CSIRO) Environment, Hobart, TAS 7004, Australia; ^e^Ecology and Conservation in the Anthropocene (ECOA) Lab, Instituto de Ciências Biológicas e da Saúde, Universidade Federal de Alagoas, Maceió 57072-900, Alagoas, Brazil

**Keywords:** macroplastic, ingestion, risk assessment

## Abstract

Plastic ingestion has been documented in nearly 1,300 marine species, including every seabird family, marine mammal family, and sea turtle species. Acute mortality, due to obstruction, perforation, or torsion of the gastrointestinal (GI) tract, has been confirmed via necropsy in all three taxa; however, quantitative risk assessment for macroplastic ingestion poses unique challenges, with risk more dependent on probability of discrete events involving diverse plastic types rather than cumulative exposure models (e.g., LC_50_). We model mortality risk associated with macroplastic ingestion in seabirds, marine mammals, and sea turtles, using data from more than 10,000 necropsies reported in the academic literature and stranding network databases. Employing an adapted Weibull Accelerated Failure Time model, we assess the relationship between the GI load (pieces and volume/animal length) of different plastic types—hard, soft, rubber, or fishing debris—and likelihood of plastic-induced mortality. Overall, 35% of seabirds, 12% of marine mammals, and 47% of sea turtles ingested plastic, and 1.6%, 0.7%, and 4.4% died from plastic, respectively. When modeling plastic together, a 90% chance of mortality was associated with 23 pieces (0.098 cm^3^/cm) in seabirds, 29 pieces (39.89 cm^3^/cm) in marine mammals, and 405 pieces (5.52 cm^3^/cm) in sea turtles (377 for juveniles). The plastic types that posed the greatest risks were rubber for seabirds, soft plastics and fishing debris for marine mammals, and hard and soft plastics for sea turtles. This research furthers scientific understanding of the likelihood of mortality from plastic ingestion and can inform monitoring, risk assessments, and management frameworks.

## Significance

Plastic ingestion is a known cause of mortality across taxa, yet the quantitative risk plastic ingestion poses is still poorly understood. Based on data from more than 10,000 necropsies, we estimate the likelihood of mortality due to the gastrointestinal load of various plastic materials—hard, soft, rubber, and fishing debris—for seabirds, marine mammals, and sea turtles. We find that 6 to 405 pieces of ingested macroplastic (or a volume between 0.044 and 39.89 cm^3^/cm body length) lead to a 90% chance of mortality in these marine species. Importantly, the amount varies depending on plastic types ingested and taxon. Our findings can be used to better understand the mortality risk of macroplastic pollution and inform future risk assessment frameworks.

Plastic pollution is pervasive in the marine environment, with plastic ingestion documented in nearly 1,300 marine species (1, 2). Ingestion of macroplastic has been linked to mortality in all marine vertebrate classes—fishes, birds, mammals, and reptiles (1, 3). Concerned by this growing body of research, scientists and governments are calling for the development of appropriate plastic monitoring and risk assessment frameworks, including thresholds to help inform the urgency and scale of mitigation strategies required for marine plastic pollution (4–6). Quantitative risk assessments, which are commonly used to inform regulatory benchmarks, offer a systematic approach for linking exposure (or amount) to the effect of a given pollutant on organisms, populations, species, or ecosystems (7, 8).

Despite broad interest and recent advancements in developing risk assessments for both microplastics (8, 9) and macroplastics (10, 11), applying traditional frameworks is not as straightforward as it is for other pollutants. For microplastics, difficulty lies in understanding how to best characterize and represent the diverse mixture of particles in risk assessments (12). Example decision-points researchers are faced with for model development include the following: 1) Should a risk assessment include the full mixture, or be relevant to different material types? and 2) What particle characteristics—particle volume, surface area, length, or shape—should inform the exposure parameter (13, 14)? Like microplastics, macroplastics also represent a broad suite of heterogeneous materials with a variety of characteristics (e.g., chemical composition, shape, size) that influence both ingestion rates and likelihood of mortality if consumed (15, 16). They may also influence the mechanism of effect (13). For example, consumption of both macro- and microplastics may lead to deleterious health effects in animals due to food dilution/starvation (17, 18), injury (19 –21), or fibrosis (22).

Quantifying the risk of macroplastic ingestion also poses unique challenges compared to microplastics (10, 23). While laboratory studies can be used to inform quantitative risk assessments for microplastics (e.g., to determine an LC_50_), it is difficult and often unethical to conduct laboratory or manipulative experiments to measure the effects of macroplastic pollution (10). Additionally, traditional dose–response approaches cannot be easily applied to understand the physical risks plastic pollution poses to marine megafauna, because likelihood of death from ingestion is determined by the probability of a fatality event occurring due to plastic consumed rather than cumulative exposure (20, 23, 24). Quantifying animal death from macroplastic ingestion and assessing cause of death are usually determined via necropsies of animals washed up on shorelines. From these, acute mortality is most often observed due to injury of the gastrointestinal (GI) tract (25, 26). In these instances, it is hypothesized that the shape, size, and other characteristics of the plastic likely influence the probability of a negative outcome, such as injury or death (27, 28).

Threshold-based risk assessments have two primary components: exposure (to a measured amount of contaminant) and impact (hazard value). Together, these inform the relationship between pollutant dose and the physiological or physical harm it poses (10, 24). To build a risk assessment for macroplastic ingestion, the exposure component should consider the likelihood of ingestion based on environmental concentrations; however, this is complicated both by data deficiencies for measuring plastic in the environment (especially the open ocean) and the diversity of the type of plastic to which an animal is exposed. To date, several methods have been developed to estimate likelihood of ingestion based on predicted environmental concentrations of plastic (often diverse items grouped together), frequency of occurrence (i.e., % of individuals that ingested plastic), and species traits (29, 30). Little research has been conducted on the impact component of macroplastic pollution, which focuses on the likelihood of mortality due to ingestion and accounts for a diversity of plastic items (24, 31).

Estimates for lethal ingestion thresholds have been developed for sea turtles and Procellariiform seabirds using the GI load of the animal paired with a demonstrated effect outcome (20, 32). These studies provide important foundations for how mortality risk can be estimated; however, they do possess significant limitations. First, these extant models assume all pieces of plastic pose equal risk, regardless of their size or material characteristics. Second, they employ only localized datasets (e.g., location-specific). Both the animals—species and age classes—and types of plastic encountered vary by location (33, 34). Therefore, thresholds based on local datasets will not necessarily be representative of mortality risk across geographies.

In this study, we aim to quantify the likelihood of acute mortality for seabirds, marine mammals, and sea turtles, based on the amount (count and volume/cm body length) and type of plastic in their GI tract. We first present a comprehensive review of the literature on mortality of seabirds, marine mammals, and sea turtles to identify reported amounts and types of plastic quantified in individual animals and mortality due to plastic ingestion for these three taxa. We then adapt a Weibull Accelerated Failure Time (Weibull AFT) model to estimate mortality risk for each taxon based on the number, as well as the volume/animal length (cm), of macroplastics (≥5 mm) of different types found in their GI tracts. To capture the differential risk posed by different types and sizes, we consider four categories of plastic—hard, soft, rubber, fishing debris (i.e., line, rope, and nets). Our findings can be paired with previous or future efforts to measure exposure to build coordinated risk assessment and management frameworks that can be informed by monitoring programs to reduce plastic pollution.

## Methods

Seabirds, marine mammals, and sea turtles were the three major taxa included in this analysis. These taxa were selected because mortality from macroplastic ingestion due to acute injury of the GI tract has been documented in all three groups (35–38). To develop our mortality models (*SI Appendix*, Fig. S1*A*), we conducted a systematic review of the peer-reviewed literature and searched stranding network databases to collect necropsy data. After collating our dataset, we conducted a meta-analysis to quantify and characterize the current state of knowledge on acute mortality from macroplastic ingestion. We then identified the taxa-plastic type combinations (e.g., marine mammals and soft plastics ingested) that were data-sufficient for modeling. Finally, we developed mortality models to identify the percent chance of mortality for an individual animal based on the burden (number of plastic pieces and volume) in its GI tract. As examples, we share the thresholds for where our model predicts an organism has either a 50% or 90% likelihood of mortality based on the load of a given plastic type in its GI tract. Here, we only focus on acute mortality due to plastic ingestion; however, entanglement in macroplastics is another driver of acute mortality in marine organisms and may even be more lethal than ingestion (11, 39, 40).

Furthermore, chronic and sublethal effects of macroplastic ingestion (e.g., food dilution) may contribute to mortality (17). Macroplastic ingestion has been correlated with poor body condition in necropsied animals; however, poor body condition cannot be attributed to plastic ingestion from necropsies alone, as poor body condition is also a risk factor that increases likelihood of ingesting macroplastics (26). As a result, further research is required to include these effects in mortality thresholds for macroplastic ingestion.

### Data Collection.

We collected necropsy data to inform our model from peer-reviewed literature published between the years 1900 and June 2023. We began with the literature review presented in ref. 23—which included data from 1900 to March 2021—and expanded it through December 2023 using the Web of Science Core Collection database. We used five search queries: “plastic debris AND ingestion,” “macroplastic AND ingestion,” “mesoplastic AND ingestion,” “marine debris AND ingestion,” “fishing debris AND ingestion.” To ensure data from all relevant papers were captured, we used the same queries in the Scopus database, and finally, cross-checked our publication list with the literature presented in the GLOVE database (2). Next, we independently searched national stranding network databases from all known networks in the United States or any database mentioned in the peer-reviewed literature (See *SI Appendix,* Fig. S1*B* for our PRISMA diagram).

For inclusion, titles and abstracts were assessed to ensure the paper presented original data on plastic ingestion in seabirds, marine mammals, or sea turtles. Next, each study was read to see whether it contained both ingestion and necropsy data that could inform our models. To be included, necropsy data on the GI tract (for turtles and mammals mouth to anus, and for birds the proventriculus and gizzard) was required at the level of an individual organism, with information on the pieces and sizes of the plastic consumed by plastic type—hard, soft (e.g., film), fishing debris (i.e., line, rope, or net), rubber, foam, cloth, or other (e.g., hose pipe, plastic burlap). Individuals from selected studies that did not ingest any plastic were included in the dataset as well. Additionally, studies needed to identify for each animal: 1) if plastic ingestion was or was not the cause of death (studies that explicitly stated none of the individuals died from plastic ingestion were included); and 2) when plastic was the cause of death, what plastic type (or types) was (or were) responsible. In instances where the minimum required data were not identified in the published material, but the analyses suggested the required data were collected, corresponding authors were contacted, and data were requested. Additional new data were included from two of the authors whose previously published necropsy data were identified in the literature review. Specimen collection and necropsy methodologies for new individuals followed the methodologies described in their previously published work (20, 41).

For the purposes of this study, the causes of plastic mortality evaluated were those linked to acute physical impacts in the GI tract—specifically perforation, obstruction, and torsion. Perforation refers to the puncturing of the GI tract (20, 42, 43). Foreign body obstruction (hereafter, “obstruction”) refers to a blockage of the GI tract with plastic material that prevents the movement of food through the GI tract (26, 44, 45). Finally, torsion refers to the twisting of the intestines, which again is a form of obstruction that prevents the movement of ingested material through the GI tract (28). A few studies attributed mortality to plastic ingestion based on emaciation and large amounts of plastic in the body as evidence for a plastic-caused death; however, we excluded these animals because the cause of death was not fully certain. These animals were excluded out of an abundance of caution as not to overinflate plastic deaths and as previously noted a causal link between food dilution and mortality is difficult to establish in beach-washed animals without clear, lethal harm identified in the GI tract (26).

For each animal that was included, we compiled data from the publication on its species, family, age, sex, individual size, species size, location of collection, and cause of death. When species and age class were given without body size, we estimated body size based on the mean size for a given age class and species. When body size and species were given without age class, we categorized age class based on mean size ranges for each life stage of a species. We also included data on the plastic they consumed: the number of pieces (including zeros from these studies) and volume by plastic category, and if applicable, the plastic type or item that caused death. When detection limits were below 5 mm (e.g., 1 mm), we excluded all pieces smaller than 5 mm in one dimension from our analysis. When detection limits were not provided, we assumed all macroplastics (>5 mm) were captured. Cause of death categories included: “known not debris” death (KND), “known debris” deaths (KD) or “probable debris” deaths (PD), and “indeterminate deaths” (IND). Volume was estimated based on data availability using the methods described in the decision tree (*SI Appendix,* Fig. S2).

### Ingestion and Mortality Rates by Taxon and Plastic Type.

First, we explored the frequency of ingestion for different material types across taxa, which provides some insight into their risk of exposure. We conducted a Non-Metric Multidimensional Scaling (NMDS) analysis to assess variations in plastic communities within the GI tracts of different taxa and a PERMANOVA test to determine whether plastic compositions differed significantly by taxa. These were both done using the vegan package in R Version 4.4.1, with a Bray–Curtis dissimilarity used as the distance measure for the NMDS.

We then evaluated frequency of occurrence, a binary measure of the percentage of each taxon that consumed each plastic type, by coding all individuals by yes (consumed >0 pieces) or no (consumed 0 pieces) based on whether they ingested each type of plastic. Using these values, we conducted χ-squared tests or Fisher’s exact tests for each taxon, depending on the sample size and distribution for each analysis (test used is specified in the results, *P* < 0.05). Statistically significant results indicate that the organisms within each taxon ingested different plastic types at different frequencies.

Finally, we explored whether the number of individuals who died from ingesting plastic varied by plastic type. We partitioned the data to include only the individuals from each taxon who consumed any amount (>0) of a given plastic type, then coded them as yes or no depending on whether the authors had determined that the animal died from ingesting that material type or not. Again, we conducted χ-squared tests or Fisher’s exact tests for each taxon, depending on the sample size and distribution for each analysis (test used is specified in the results, *P* < 0.05). Statistically significant results indicated that the chance of mortality if a given item was consumed varied by plastic type within the taxon. All analyses were conducted in R Version 4.4.1 (See open access R code in *SI Appendix*).

### Mortality Model Development.

#### Identifying taxon-plastic combinations to model.

First, we identified the number of animals in our dataset that ingested each type of plastic, the types of plastics they ingested, and the relationship between ingested plastics and cause of death. To be data sufficient for developing a type-specific model, at least five individual animals had to have died from ingestion of that plastic type. Next, we evaluated each taxon-plastic category combination (e.g., seabirds and rubber ingestion) to identify data-sufficient combinations for quantifying mortality risk. To do this, we conducted Kruskal–Wallis and Dunn tests comparing the amount of plastic ingested by animals in the KND, KD or PD, and IND categories. These nonparametric tests were used to identify any statistically significant differences between groups, and then calculate pair-wise differences between groups, respectively. Animals in the KD category needed to have significantly higher plastic ingestion loads than those in the KND category, with animals in the IND category falling in between the two groups (*SI Appendix*, Figs. S4–S6). We combined PD and KD deaths into one category because relatively few PD deaths occurred, and each showed evidence of acute, severe harm to the GI tract.

After applying these criteria, we were able to develop mortality models for all three taxa for specific materials. For seabirds, we modeled the likelihood of mortality due to total plastic ingestion, hard plastic, and rubber. For marine mammals, we modeled the likelihood of mortality due to total plastic ingestion, soft plastics, and fishing debris, and for sea turtles, we modeled total plastic ingestion, hard plastics, and soft plastics. We also modeled total plastic ingestion for juvenile sea turtles in their oceanic life stage or recently recruited to the neritic zone [<35 cm curved carapace length (CCL)].

#### Modeling likelihood of ingested plastic mortality.

To develop each mortality risk model, we adapted a Weibull AFT model to assess the relationship between plastic exposure (load in the GI tract) and the likelihood of mortality. Although, AFT models are typically used in survival analyses to estimate the time until a health-related event occurs (e.g., death) (46), they have also been adapted and applied to model the relationship between exposure concentrations and adverse health outcomes, such as allergen load and allergic reaction, in place of time (47). We selected the AFT framework over previously applied logistic regression models (20, 32), because it allows for the incorporation of right censored individuals—individuals observed with a plastic load that did not lead to death. Right censoring enables these individuals to be included in the model in a statistically appropriate way, acknowledging that their true failure threshold remains unknown and that they should inform the model differently than individuals who experienced failure (i.e., death from plastic) (48). We selected a Weibull distribution, based on its fit to our data (*SI Appendix*, Figs. S7–S9).

[1] $$\mathrm{Probability}\;\mathrm{of}\;\mathrm{death}=1-e^{-{\left(\frac{p}{e^{\beta_{0}+\beta_{1}x_{1}+\beta_{n}x_{n}}}\right)}^{\frac{1}{\sigma }}}.$$

The cumulative incidence function of the Weibull AFT (Eq. **1**). can be used to estimate the likelihood of death given predictor variables, where *p* is the plastic load. In our models, KD and PD deaths were coded as the failure point, or death from plastic. Individuals who did not die from plastic (KND) were right censored at the level of plastic they had in their gut. We excluded IND deaths from our analysis due to the uncertainty they contributed to the model.

For each taxon-plastic category combination, we ran both simple univariate models and multivariate models. The simple univariate models considered likelihood of KD based on the load of plastic [# of pieces or volume (cm^3^)/animal length (cm)] in an individual’s GI tract. We chose to look at volume as it relates to body length (species body length for seabirds, individual CCL for sea turtles, and individual body length for marine mammals) given that the volume of plastic we anticipate will cause harm is directly related to the size of an individual’s GI tract. We use body length as a proxy for GI tract volume, given for most species the volume of the GI tract is not known, and body size and gut length are strongly correlated within these taxa (49–53). The multivariate models considered other predictor variables including individual size and average size for the species. Other predictor variables including species, family, and age class were explored; however, data availability did not allow for their inclusion. We used bootstrapping with 1,000 iterations for each model to estimate the 95% CI.

To evaluate model selection and performance for the best fit predictive model for each taxon-material combination, we evaluated the Akaike Information Criterion (AIC), as well as the *P*-value and SE for added variables. Alternative models along with the AIC values and parameter values are provided in *SI Appendix*, Table S1.

After choosing the best fit for each taxon-plastic category model, we then graphed the cumulative distribution functions, using mean parameter estimates, to show the probability of death based on the amount of plastic consumed, along with the 95% CI. We also present the predicted values for the plastic load, by plastic type, that will lead to a 50% chance and 90% chance of mortality in each taxon. All mortality modeling and subsequent analyses were conducted in R Version 4.4.1 using the survival package (See open access R code in *SI Appendix*).

## Results

### Data at a Glance.

**Fig. 1.**
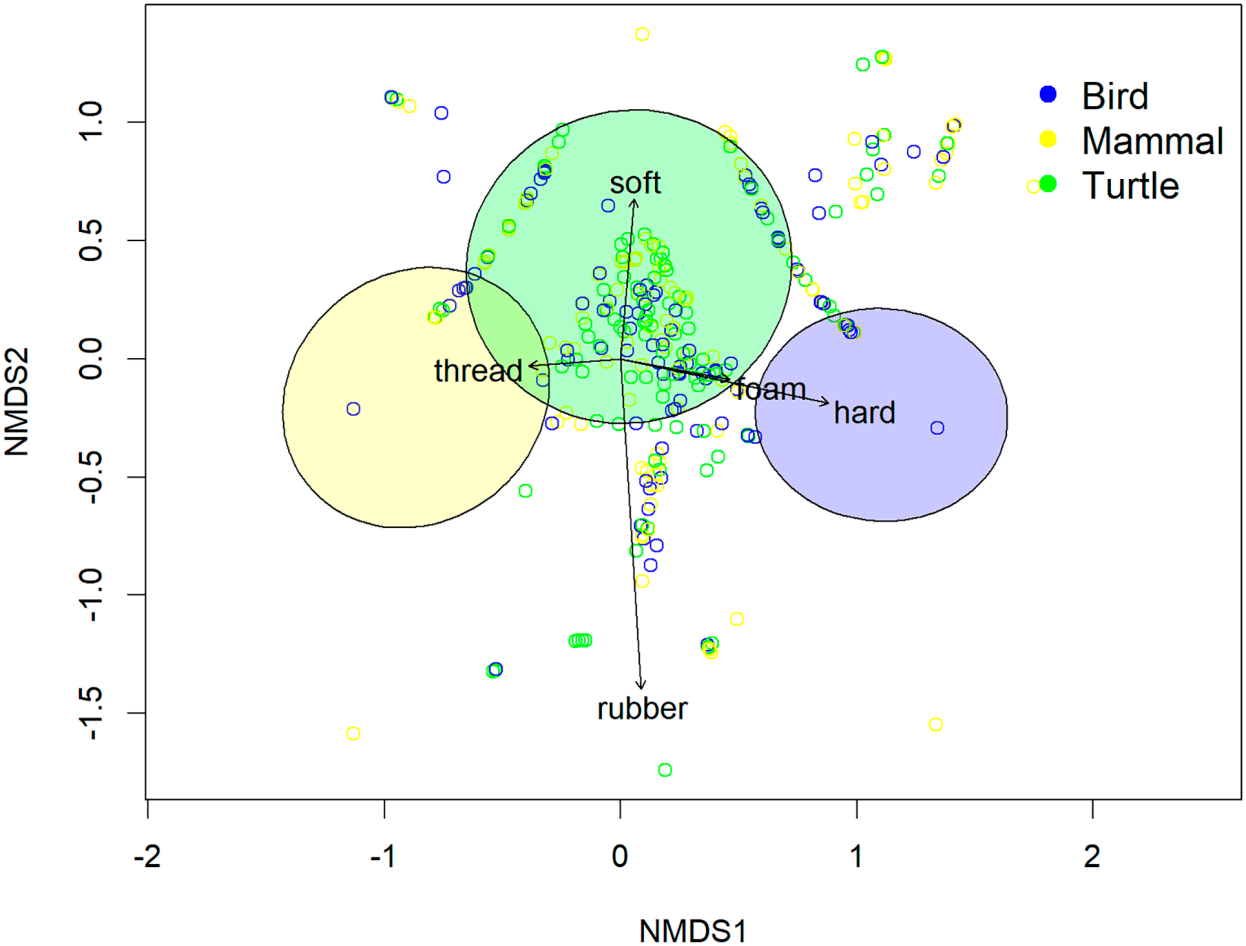
NMDS plot showing the different communities or types of macroplastic consumed by the three taxa included in our study (sea birds, sea turtles, and marine mammals). Each taxon consumed distinct communities of macroplastic. The circles on the graph are centered around the mean, and the size represents one SD of point scores for each taxon.

We collected necropsy data for 10,412 individuals from 57 sources [53 peer-reviewed publications, the NOAA Fisheries stranding network database (54), the FWC Fish and Wildlife Research Institute manatee mortality database (55), and two new datasets (*SI Appendix*)]. Of the individuals, 1,537 were seabirds (17 families, 57 species), 7,569 were marine mammals (10 families, 31 species), and 1,306 were sea turtles (seven species). The proportion of different plastic types found in the GI tract of individuals varied by taxon (Fig. 1, PERMANOVA: *P* < 0.001).

Of the seabirds in our dataset, 35.4% consumed macroplastic (*SI Appendix*, Table S4 contains species-specific ingestion rates), but the frequencies of ingestion for different material types were significantly different (χ-squared: *P* < 0.001, Table 1 and *SI Appendix,* Fig. S3*A*). For all plastic types, most seabirds that consumed a plastic type only contained one piece of that type, and hard plastics were the only type where one individual contained more than 10 pieces (*SI Appendix,* Fig. S3*C*). Nearly all individuals who ingested a plastic type contained less than 1 cm^3^ (mean piece size = 0.046 cm^3^) of the material within their GI tract (*SI Appendix,* Fig. S3*D*). Of the seabirds that consumed plastics, 3.1% were categorized as KD, and an additional 1.5% as PD (*SI Appendix,* Fig. S3*B*). Likelihood of death differed significantly by the ingested plastic type (Table 1, Fisher’s Exact Test: *P* < 0.016), with the highest proportion of plastic death for seabirds being from rubber (Table 1). Other causes of death for seabirds included fisheries interaction (16.0%), culled for research (8.5%), starvation (2.1%), and disease (0.8%), with most studies only identifying if plastic was or was not the cause of death (unreported 70.9%).

**Table 1.** Plastic ingestion and mortality rates

Taxa	Hard	Soft	Fishing debris	Rubber	Foam	Cloth	Total
Animals that consumed each plastic type (%)							
Seabirds	32.2	3.3	2.9	2.1	1.6	0	35.4
Marine mammals	0.3	1.2	8.6	0.6	0.2	0.09	11.9
Sea turtles	19.7	32.3	27.4	1.9	3.3	0.6	47.3
Animals that died of ingestion out of those that consumed plastic (KD%/PD%)							
Seabirds	2.0/0.8	0/1.96	2.2/2.2	15.2/6.1	0/0	–	3.1/1.5
Marine mammals	4.8/0	12.6/2.3	4.3/0.3	0/0	0/0	0/0	5.0/0.5
Sea turtles	4.3/0.8	4.5/0.5	1.4/0.3	4.0/0	0/0	0/0	8.4/0.8

KD is known debris death, PD is probable debris death.

Of the marine mammals in our dataset, 11.9% consumed macroplastics, but the frequency of ingestion for different material types was significantly different (Fisher’s Exact Test: *P* < 0.001, Table 1 and *SI Appendix,* Fig. S3*E*). Most marine mammals that consumed a plastic type only contained one piece; fishing debris and soft plastics being the plastic types most frequently observed more than once (*SI Appendix,* Fig. S3*G*). Individuals contained a large range in plastic volume (mean piece size = 164.85 cm^3^), with many individuals consuming more than 10 cm^3^ of a given material type (*SI Appendix,* Fig. S3*H*). Of the marine mammals that consumed plastics, 5% were categorized as KD, and an additional 0.5% as PD (*SI Appendix,* Fig. S3*F*). Likelihood of death by plastic type for individuals that consumed plastic differed significantly by material (Fisher’s Exact Test: *P* = 0.006), with the highest proportion of plastic death for marine mammals being from soft plastic (Table 1). Other causes of death for marine mammals included vessel strike (12.7%), cold stress (10.2%), red tide (8.4%), fisheries interaction (2.69%), canal locks (0.6%), young abandonment (0.3%), culled for research (0.2%), and mass strandings (0.2%), with most studies only identifying if plastic was or was not the cause of death (unreported 63.7%). Additional causes of death reported accounted for less than 0.1% of deaths (animal interactions, barotrauma, disease, starvation, nonplastic ingestion, and sonar).

Of the sea turtles in our dataset, 47.3% of individuals consumed macroplastic, and the frequency of ingestion for different types was significantly different (χ-squared: *P* < 0.001, Table 1 and *SI Appendix,* Fig. S3*I*). Most individuals (>50%) who ingested a plastic type ingested multiple pieces, with many individuals containing over 10 pieces of hard plastics, soft plastics, and fishing debris (*SI Appendix,* Fig. S3*K*). Still, the volume of these pieces was generally small (mean piece size = 0.22 cm^3^) with more than half of individuals containing less than 1 cm^3^ of a given material type, and nearly all individuals containing less than 10 cm^3^ of any plastic type (*SI Appendix,* Fig. S3*L*). Of the sea turtles that consumed plastics, 8.4% were categorized as KD, and an additional 0.8% were categorized as PD (*SI Appendix,* Fig. S3*J*). Likelihood of death by plastic type for individual sea turtles that consumed plastics did not differ significantly by plastic type (Fisher’s Exact Test: *P* = 0.10, Table 1). Other causes of death for sea turtles included fisheries interaction (16.1%), disease (1.6%), nonplastic debris ingestion (1.1%), and vessel strike (0.2%), with most studies only identifying if plastic was or was not the cause of death (unreported 76.7%).

### Model Outputs.

#### Seabirds.

**Fig. 2.**
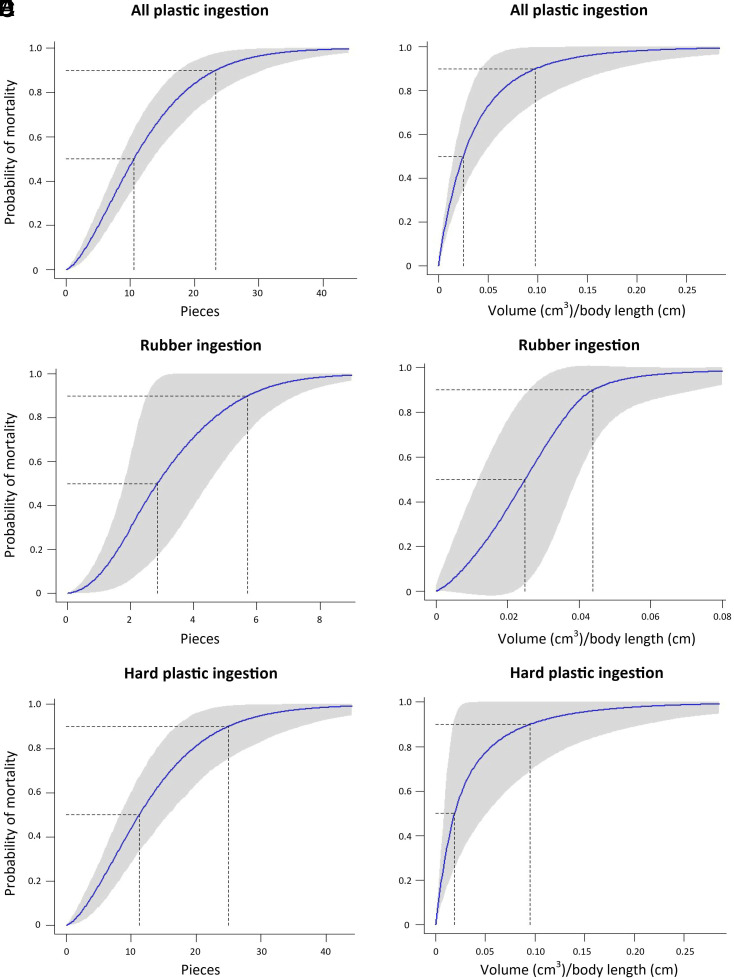
Curves showing probability of mortality based on the amount of macroplastic in the GI tracts of seabirds. (*A*) Total pieces of macroplastic, (*B*) Volume of macroplastic/body length, (*C*) Pieces of rubber, (*D*) Volume of rubber/body length, (*E*) Pieces of hard macroplastic, (*F*) Volume of hard macroplastic/body length.

For seabirds, the simplest models proved to be the best fit for the data. When all plastic types were considered, a seabird with a plastic load of 11 pieces (95% CI: 8–13, N = 152) ≥5 mm in length or 0.025 cm^3^/cm body length (95% CI: 0.015–0.044 cm^3^/cm, N = 131) had a 50% chance of mortality (Table 2). A seabird with a plastic load of 23 pieces (95% CI: 18–29) ≥5 mm in length or 0.098 cm^3^/cm (95% CI: 0.043–0.18 cm^3^/cm) had a 90% chance of mortality (Fig. 2 *A* and *B*). Table 3 provides the volume thresholds for the smallest and largest bird species in our dataset.

**Table 2.** Amount of ingested macroplastic associated with a 50% and 90% likelihood of mortality for each taxon studied (sea birds, sea turtles, and marine mammals)

Mortality model	50% threshold (#)	50% threshold (size)	90% threshold (#)	90% threshold (size)
Seabirds				
Total plastic	11 pieces	0.025 cm^3^/cm	23 pieces	0.098 cm^3^/cm
Hard plastic	11 pieces	0.019 cm^3^/cm	25 pieces	0.095 cm^3^/cm
Rubber	3 pieces	0.025 cm^3^/cm	6 pieces	0.044 cm^3^/cm
Marine mammals				
Total plastic	12 pieces	4.71 cm^3^/cm	29 pieces	39.89 cm^3^/cm
Soft plastic	12 pieces	0.77 cm^3^/cm	29 pieces	4.51 cm^3^/cm
Fishing Debris	10 pieces	2.93 cm/cm	28 pieces	8.73 cm/cm
Sea turtles (all, including <35 CCL)				
Total plastic	118 pieces	0.91 cm^3^/cm	405 pieces	5.52 cm^3^/cm
Hard plastic	NA	0.66 cm^3^/cm	NA	2.88 cm^3^/cm
Soft plastic	93 pieces	NA	342 pieces	NA
Young sea turtles (<35 CCL)				
Total plastic	105 pieces	–	377 pieces	–

These thresholds varied by plastic material type. “NA” indicates where data were insufficient to inform threshold. Size thresholds are volume (cm^3^) or, for fishing debris, length (cm)/cm body length (CCL for sea turtles). CCL= curved carapace length.

**Table 3.** Absolute volume thresholds for the smallest and largest adult individuals from our dataset based on the size thresholds reported in Table 2

Seabird absolute volume thresholds							
Species	Length (cm)	Total 50%	Total 90%	Hard 50%	Hard 90%	Rubber 50%	Rubber 90%
Pelecanoides georgicus	19.5	0.49 cm^3^	1.91 cm^3^	0.37 cm^3^	1.85 cm^3^	0.49 cm^3^	0.86 cm^3^
Diomedea exulans	121.5	3.04 cm^3^	11.91 cm^3^	2.31 cm^3^	11.54 cm^3^	3.04 cm^3^	5.35 cm^3^
Marine mammal absolute volume thresholds (length for fishing debris (FD))							
Species	Length (cm)	Total 50%	Total 90%	Soft 50%	Soft 90%	FD 50%	FD 90%
Phocoena phocoena	122.5	576.98 cm^3^	4,886.53 cm^3^	94.33 cm^3^	552.48 cm^3^	358.93 cm	1,069.43 cm
Balaenoptera physalus	1,935	9,113.85 cm^3^	77,187.15 cm^3^	1,489.95 cm^3^	8,726.85 cm^3^	5,669.55 cm	16,892.55 cm
Sea turtle absolute volume thresholds							
Species	CCL (cm)	Total 50%	Total 90%	Hard 50%	Hard 90%		
Lepidochelys kempii (adult)	56.7	51.60 cm^3^	312.98 cm^3^	37.42 cm^3^	163.30 cm^3^		
Dermochelys coriacea (adult)	156	141.96 cm^3^	861.12 cm^3^	102.96 cm^3^	449.28 cm^3^		
*Chelonia mydas* (posthatchling)	5.2	4.73 cm^3^	28.70 cm^3^	3.43 cm^3^	14.98 cm^3^		
*Chelonia mydas* (juvenile)	35	31.85 cm^3^	193.20 cm^3^	23.10 cm^3^	100.80 cm^3^		

When only considering rubber ingestion, a seabird with a rubber load of three pieces (95% CI: 2–4, N = 17) ≥5 mm in length or 0.025 cm^3^/cm (95% CI: 0.012–0.037 cm^3^/cm, N = 13) had a 50% chance of mortality (Fig. 2 *C* and *D*). A seabird with a rubber load of six pieces (95% CI: 3–7) ≥5 mm in length or 0.044 cm^3^/cm (95% CI: 0.025–0.073 cm^3^/cm) had a 90% chance of mortality.

When only considering hard plastic ingestion, a seabird with a plastic load of 11 pieces (95% CI: 8–16, N = 107) ≥5 mm in length or 0.019 cm^3^/cm (95% CI: 0.0083–0.052 cm^3^/cm, N = 96) had a 50% chance of mortality (Fig. 2 *E* and *F*), and a seabird with a hard plastic load of 25 pieces (95% CI: 17–36) ≥5 mm in length or 0.095 cm^3^/cm (95% CI: 0.018–0.21 cm^3^/cm) had a 90% chance of mortality (See *SI Appendix*, Table S1 for alternative models).

#### Marine mammals.

**Fig. 3.**
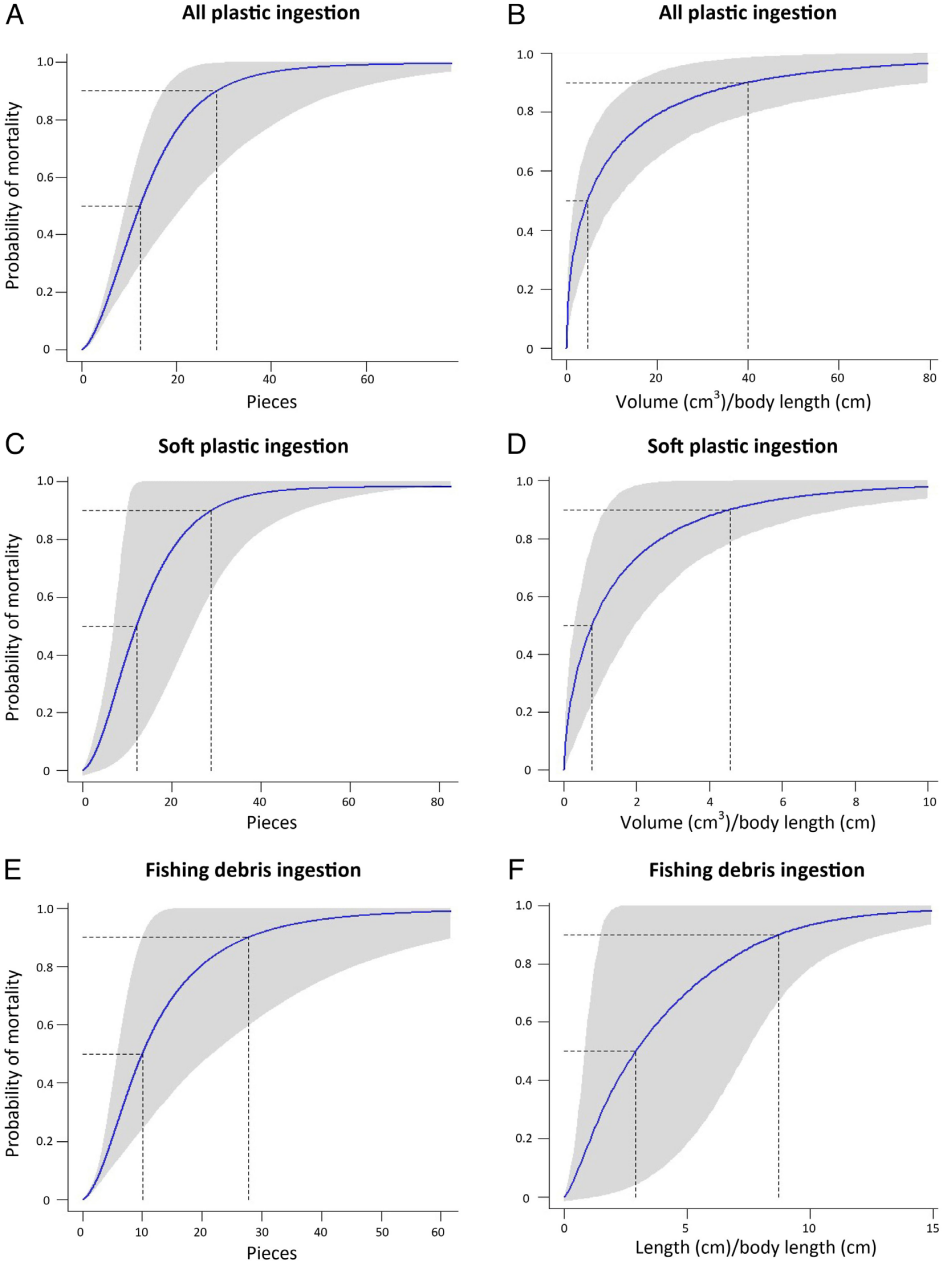
Curves showing probability of mortality based on the amount of macroplastic in the GI tracts of marine mammals. (*A*) Total pieces of macroplastic, (*B*) Total volume of macroplastic/body length, (*C*) Pieces of soft macroplastic, (*D*) Volume of soft macroplastic/body length, (*E*) Pieces of fishing debris, and (*F*) Length of fishing debris/body length.

For marine mammals, the best fit models for all and soft plastic pieces included species size, while the best fit model for fishing debris included individual size. When all plastic types were considered, a marine mammal with a plastic load of 12 pieces (95% CI: 9–21, N = 895) ≥5 mm in length or 4.71 cm^3^/cm body length (95% CI: 1.84–10.98 cm^3^/cm, N = 76) had a 50% chance of mortality (Table 2). A marine mammal with a plastic load of 29 pieces (95% CI: 17–59) ≥5 mm in length or 39.89 cm^3^/cm (95% CI: 15.09–80.21 cm^3^/cm) had a 90% chance of mortality (Fig. 3 *A* and *B*). Table 3 provides the volume thresholds for the smallest and largest marine mammal species in our dataset.

When only considering soft plastic ingestion, a marine mammal with a plastic load of 12 pieces (95% CI: 7–22, N = 83) ≥5 mm in length or 0.77 cm^3^/cm (95% CI: 0.29–1.93 cm^3^/cm, N = 40) had a 50% chance of mortality (Fig. 3 *C* and *D*). A marine mammal with a soft plastic load of 29 pieces (95% CI: 11–48) ≥5 mm in length or 4.51 cm^3^/cm (95% CI: 1.12–7.76 cm^3^/cm) had a 90% chance of mortality.

When only considering fishing debris ingestion, a marine mammal with a load of 10 pieces (95% CI: 6–22, N = 644) ≥5 mm in length or a length of 2.93 cm/cm body length (95% CI: 0.84–7.40, N = 35) had a 50% chance of mortality (Fig. 3 *E* and *F*). A marine mammal with a fishing debris load of 28 pieces (95% CI: 10–63) ≥5 mm in length or a total length of 8.73 cm/cm body length (95% CI: –7.60) had a 90% chance of mortality.

#### Sea turtles.

**Fig. 4.**
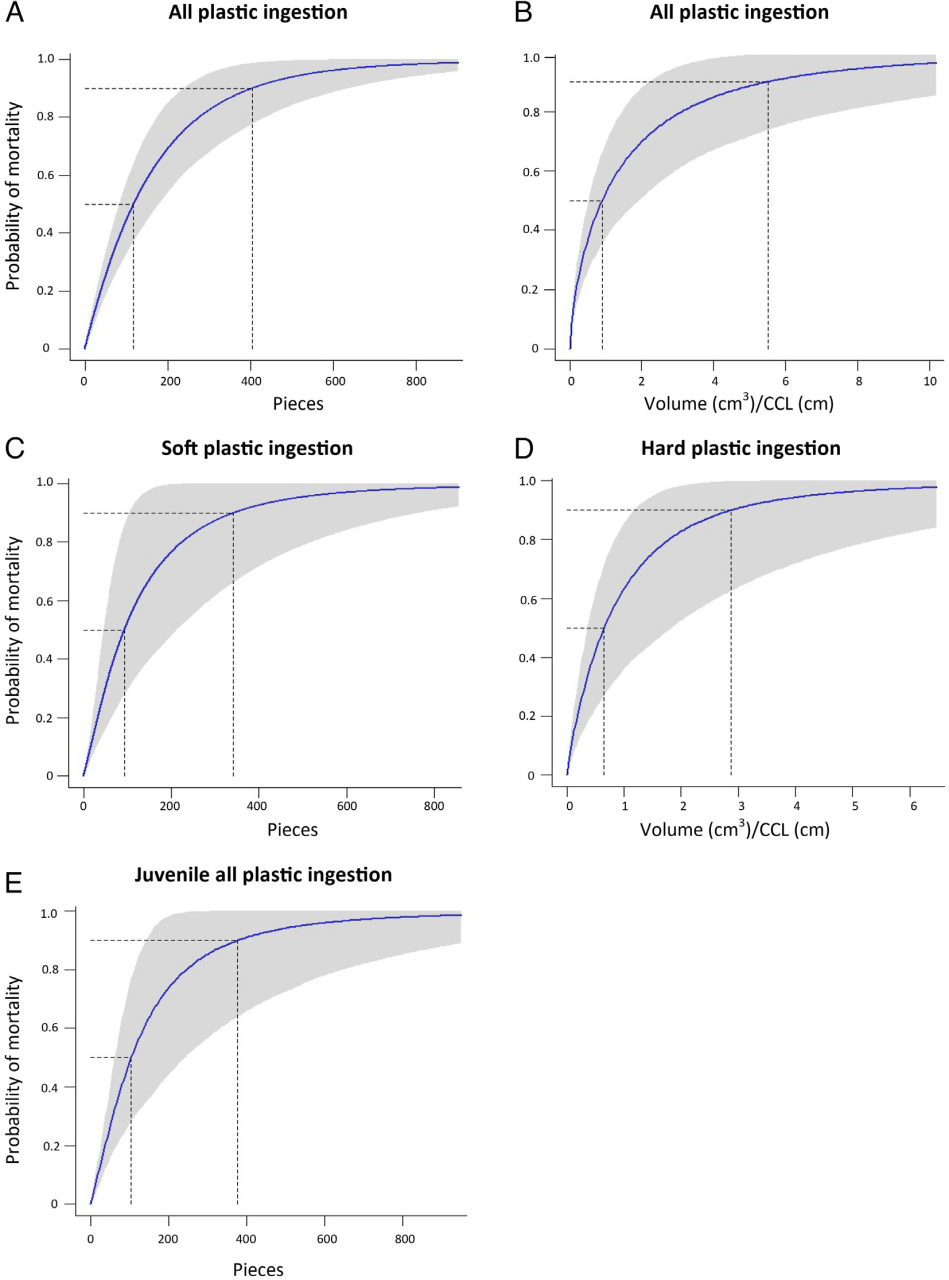
Curves showing probability of mortality based on the amount of macroplastic in the GI tracts of sea turtles. (*A*) Total pieces of macroplastic, (*B*) Total volume of macroplastic/CCL, (*C*) Pieces of soft macroplastic, (*D*) Volume of hard macroplastic/CCL, (*E*) Juvenile turtle (<35 CCL) all macroplastic ingestion. CCL= curved carapace length.

For sea turtles, the best fit models for total and soft plastic pieces included individual size, while the best fit model for young turtles included species size. When all plastic types were considered, a sea turtle with a plastic load of 118 pieces (95% CI: 84–180, N = 523) ≥5 mm in length or 0.91 cm^3^/cm CCL (95% CI: 0.52–1.93 cm^3^/cm, N = 408) had a 50% chance of mortality (Table 2). A sea turtle with a plastic load of 405 pieces (95% CI: 240–640) ≥5 mm in length or 5.52 cm^3^/cm (95% CI: 2.21–14 cm^3^/cm) had a 90% chance of mortality (Fig. 4 *A* and *B*). Table 3 provides the volume thresholds for the smallest and largest adult and juveniles sea turtles in our dataset.

When only considering hard plastic ingestion, a sea turtle with a plastic load of 0.66 cm^3^/cm (95% CI: 0.36–1.84 cm^3^/cm, N = 170) had a 50% chance of mortality, and a sea turtle with a plastic load of 2.88 cm^3^/cm (95% CI: 1.18–12.84 cm^3^/cm) had a 90% chance of mortality (Fig. 4*D*).

When only considering soft plastic ingestion, a sea turtle with a plastic load of 93 pieces (95% CI: 46–214, N = 342) ≥5 mm in length had a 50% chance of mortality and a sea turtle with a load of 342 pieces (95% CI: 105–765) ≥5 mm in length had a 90% chance of mortality (Fig. 4*C*). Finally, for small juveniles (<35 cm CCL), a sea turtle with a total plastic load of 105 pieces (95% CI: 63–243) ≥5 mm in length had a 50% chance of mortality, while a sea turtle with a load of 377 pieces (95% CI: 143–998) ≥5 mm in length had a 90% chance of mortality (Fig. 4*E*).

## Discussion

### Patterns of Mortality from Ingested Load of Plastics across Taxa.

We found high incidences of plastic ingestion across all three taxa included in this study. Sea turtles contained the highest frequencies and loads of plastic. Nearly 50% of sea turtles included in our dataset had ingested plastic, compared to 35% of seabirds and 12% of marine mammals. Sea turtles were also more likely to have suffered mortality from plastic ingestion. Among sea turtles, ingested plastic was the reported cause of death in 4.4% of all necropsied individuals—almost all posthatchlings and juveniles—compared with 1.6% of all necropsied seabirds and 0.7% of all marine mammals. However, we note that necropsy datasets do not represent a random selection of healthy animals, and caution interpreting these as global frequencies of plastic ingestion mortality in wild populations (56). Still, by including individuals that were bycaught, killed for research sampling, or in studies reporting no stranded individuals died from plastic ingestion, we expect these results represent plastic ingestion rates for individuals at time of death.

The foraging ecology and habitat use of sea turtles influence plastic ingestion (38, 57, 58). Juvenile *Chelonia mydas* (green turtles), which are well represented in our data, tend to have a more generalist diet (59), which may increase the amount and types of plastic they ingest (1). Juveniles across species generally ingest plastics at higher rates than adult sea turtles (60), as they encounter plastics at high rates (61, 62) and show less prey specificity (21, 63). However, ingestion rates by plastic type varies across life stage, with posthatchling sea turtles consuming more hard plastics and adult sea turtles consuming more fishing debris and soft plastics (e.g., film) (62). Ingestion frequency in seabirds and marine mammals was more dependent on species and plastic type (64–70). For seabirds, the high prevalence of hard plastics is well documented (20, 71, 72) and likely relates to the prevalence of persistent, positively buoyant fragments that match their prey size (73, 74). For marine mammals, species that prey on commercially fished species (e.g., dolphins) frequently encounter fishing debris (66, 75, 76), while species with other feeding strategies, such as sperm whales and baleen whales, ingested a broader range of material types (42, 77, 78). Importantly, the relatively low frequencies of ingestion in marine mammals may be influenced by differences in reporting. Most studies of sea turtles and seabirds reported all pieces greater than 5 mm in length (21, 79, 80), while marine mammals research generally focused on larger macroplastics (77, 81).

### How Much Plastic Is Too Much?

When modeling all plastic types together, a 90% chance of mortality was predicted if a seabird consumed 23 pieces of plastic (or 0.098 cm^3^/cm body length), a marine mammal consumed 29 pieces (or 39.89 cm^3^/cm body length), a sea turtle consumed 405 pieces (or 5.52 cm^3^/cm CCL), and a juvenile sea turtle (<35 cm CCL) consumed 377 pieces. Across our models, our results indicate that a plastic load between 3–118 pieces (or 0.019–4.71 cm^3^/cm body length) in any given seabird, marine mammal, or sea turtle can cause a 50% chance of mortality, and 6–405 pieces (or 0.044–39.89 cm^3^/cm body length) can cause a 90% chance of mortality. These thresholds vary due to taxa, plastic type, and, for sea turtles, life stage. Generally, plastic volume/animal size (or length for fishing debris) is a better indicator of acute mortality risk than pieces. This is because the area a material occupies in the gut compared to the gut size more directly influences how likely an item is to obstruct, perforate, or twist the GI tract. For example, 100 pieces of line 1 mm in length is unlikely to cause acute injury in an adult marine mammal; but just one piece of line that is several meters long can (82). Further, reporting biases against small pieces (i.e., detection limits >5 mm) will have greater effects on estimates of pieces than total volume. This is more likely to be an issue for large mammals, where detection limits are frequently not described (26). For example, 5 mm fragments would be a small contribution to the total volume of plastic eaten by a sperm whale compared to several large items (e.g., 5-gallon bucket, large fishing net), but their inclusion or exclusion could significantly change the reported pieces consumed (77, 82). Unfortunately, limited volume data availability prevented us from including this metric for all individuals in our dataset. In these instances where item volume data are missing and cannot be inferred, the number of pieces is still informative, because gape size and foraging behavior influence the size of plastics individuals will consume (23, 83).

The amount of plastic predicted to cause death varied dramatically by taxon. This was primarily driven by the size of the animal and the size of plastic pieces they consume. When considering all plastic types together, seabirds had the lowest thresholds (i.e., the number of pieces or volume/length associated with death was smaller than for the other taxa). The piece-based thresholds for marine mammals fell between seabirds and sea turtles. However, on average, marine mammals consumed much larger pieces of plastic (77), so volume-based mortality thresholds in marine mammals were the highest. Sea turtles had the highest mortality thresholds by pieces but mean piece size was small (0.22 cm^3^).

Overall, our results aligned with the previously reported 50% mortality threshold for seabirds, which found a similar number of total plastic pieces associated with likelihood of mortality and increased risk posed by balloons (20). However, our 50% mortality thresholds for sea turtles were much higher than previously published thresholds (32). The measured differences are probably due to two factors known to influence sea turtle mortality risk. Our global dataset included more adults, bycaught individuals, and turtles exposed to a wider range of plastic compositions, while the former focused on sea turtles that died near shore, where they are likely exposed to a different composition of plastic than those in the open ocean. Additionally, we excluded individuals with an indeterminate cause of death (IND) in our models. When considering the relative risk posed by different plastic types, our results align with previously reported expert perceptions on the materials most likely to cause ingestion mortality (84).

Within each taxon, the amount of plastic that led to mortality varied by plastic type. For example, only three pieces of rubber (mostly balloons) led to a 50% chance of mortality in seabirds. The lethality of balloons in seabirds has been attributed to their elasticity, which allows them to deform to fit internal spaces and cause obstructions at junctures in the GI tract (20). Given that seabirds may select for balloons and other rubber material when encountered (20), these items likely pose a high mortality risk to seabirds when they become marine pollution. The 50% threshold for hard plastics was higher (11 pieces) and is likely due, in part, to their smaller average size (0.040 cm^3^) and rigid shape, enabling smaller hard plastics to pass through junctures in the GI tract more easily than stretchy material (20).

For marine mammals, soft plastics and fishing debris posed a higher likelihood of mortality than plastics generally. This was largely due to obstructions from large pieces at junctures in the GI tract (85, 86). Additionally, ingesting less than 2.93 cm/cm body length of fishing debris (e.g., 293 cm for a 100 cm long individual) was associated with a 50% chance of mortality, indicating that if a marine mammal ingests just one long piece of line, net, or rope, their risk of mortality can be quite high (76, 77).

Sea turtles seemed more sensitive to soft plastics than other plastic types (62, 87). However, our results suggested that hard plastics also appeared to pose a higher mortality threat. This may be influenced by the fact that mortality from plastic ingestion was most often posthatchlings and juveniles, which may be more prone to ingesting hard plastics due to their generalist feeding habits (59, 60), and that the size range of prey for posthatchlings overlaps with the most abundant size range of buoyant hard plastics in the ocean (88, 89). Further, given the smaller gut lumen and juncture diameters in posthatchlings and juveniles compared to adults, they are more likely to die from ingestion (21).

This research can help inform future risk assessment and management frameworks by predicting the amount of plastic ingestion that may lead to mortality in seabirds, marine mammals, and sea turtles. Specifically, this risk assessment evaluated the likelihood that a given amount of plastic load in the GI tract (reported as number of plastic pieces or volume) will kill an animal. These thresholds are informed by several different effect mechanisms—obstruction, perforation, and torsion—however, they are based solely on physical effects due to acute injury. Therefore, they do not represent typical dose–response curves, and instead better reflect the probability of an acute mortality event from plastic during an individual’s life based on their average GI load (where plastics are ingested and excreted with an unknown turnover rate). Importantly, this risk assessment does not capture the total mortality risk of plastic ingestion. Plastic ingestion may also contribute to mortality through food dilution (17, 18), which was not considered. The focus on ingestion also excludes other physical effects of plastic pollution on marine wildlife. For some material, such as fishing debris, the risk of mortality due to entanglement may be higher than ingestion, particularly for some species of sea turtles and marine mammals (11, 39, 40). Therefore, this threshold cannot be understood as a comprehensive mortality threshold.

Despite limitations, these methods for modeling likelihood of mortality provide more comprehensive insights into the risk plastics pose and can be integrated into broader risk assessment and management frameworks. However, confidence in and accuracy of our model estimates rely on the quality and quantity of existing data. Most importantly, our models rely on the accuracy of mortality reporting in the published literature. Although we focused on acute mortality events to remove uncertainty associated with cause of death, improper designation of plastic mortalities could still affect the accuracy of our thresholds (e.g., nonplastic items caused observed perforations, or they occurred postmortem). Our models may also be skewed by using only dead, necropsied individuals, and thus may overrepresent mortality risk associated with certain GI loads by overrepresenting plastic deaths (57). Therefore, the opportunistic inclusion of more bycaught or culled animals in plastic ingestion datasets may provide a more refined picture of the GI loads associated with likelihood of mortality. Underreporting of plastic ingestion without negative effects will also artificially inflate the measured likelihood of mortality. Therefore, studies should clearly report whether no acute injuries were observed (57, 90). Additionally, underreporting of small macroplastics will lower the number of pieces associated with negative outcomes. Consistent size thresholds should be used to improve confidence and interpretation of model results for pieces. Standardized methodologies for reporting acute injuries should also be used to build researcher confidence in reported mortality events. Finally, many studies collect data at the individual level but only report frequency of occurrence or mean plastic number/volume for the sample population. Without providing data at the individual level, these papers cannot easily be used to inform global risk assessments.

The accuracy and precision of these mortality models and thresholds would be improved if they were further disaggregated by family, species, or age class. Modeling mortality risk for sea turtles posed challenges because risk varied so dramatically by life stage—posthatchlings died with very small GI loads, while adults were found without acute injury despite large GI loads (21, 90). With more data, mortality models could be further disaggregated and include more variables to improve their precision and ultimately, their value for management.

### Informing Risk Assessment and Management Frameworks.

This work has several important implications for advancing plastic pollution reduction policies. First, our results help elucidate the type of plastics marine organisms consume and the likelihood of mortality based on GI load. This provides insight into the ecological benefits of interventions that target important foraging areas (e.g., coastal cleanups) and problematic materials (e.g., balloon release bans or plastic bag bans). Beyond the results we present, our modeling approach can be used as a tool, adapted by decision makers to explore likelihood of mortality in their locale and updated as new data are available.

This research is also an important step forward in developing macroplastic risk assessment frameworks for marine wildlife. Risk assessments require information on both the likelihood an organism will be exposed to a potential hazard, and if exposed, the likelihood the hazard has a negative impact (10). If the relationship between environmental macroplastic concentrations and GI load can be measured, then macroplastic concentrations in the environment can be linked to mortality risk for a population. These types of risk assessments can be used by policymakers and regulators to inform environmental thresholds. Although environmental thresholds generally assume some level of pollution is inevitable and do not always ensure that no harm occurs, they do provide important regulatory levers for reducing marine pollution. Importantly, with risk thresholds being set by decision makers, they can be adapted to fit the values, goals, or needs of the regions and communities in which they are implemented. As a result, identifying quantitative thresholds is an important step in a comprehensive intervention strategy for addressing marine plastic pollution.

## Supplementary Material

Appendix 01 (PDF)

Dataset S01 (CSV)

Dataset S02 (XLSX)

Code S01 (TXT)

## Data Availability

All study data are included in the article and/or supporting information.
