# Supplementary material for: A quantitative risk assessment framework for mortality due to macroplastic ingestion in seabirds, marine mammals, and sea turtles
Source: Proc Natl Acad Sci U S A. 2025 Nov 17;122(48):e2415492122. doi: 10.1073/pnas.2415492122 (PMC12684891; doi:10.1073/pnas.2415492122)
Supplement: Supplementary file 1 — Appendix 01 (PDF) [file pnas.2415492122.sapp.pdf]

A quantitative risk assessment framework for mortality due to macroplastic ingestion in seabirds, marine mammals, and sea turtles

Authors. Erin L. Murphy[1,2*], Britta R. Baechler[1], Lauren Roman[3,4], George H. Leonard[1], Nicholas J. Mallos[1], Robson Santos[5], Chelsea M. Rochman[2]

[1]Ocean Conservancy, Washington DC, United States
[2]Department of Ecology and Evolutionary Biology, University of Toronto, Toronto, Ontario, Canada.
[3]Institute for Marine and Antarctic Studies, University of Tasmania, Hobart, Tasmania, Australia
[4]CSIRO Environment, Hobart, Tasmania, Australia
[5]ECOA Lab, Instituto de Ciências Biológicas e da Saúde, Universidade Federal de Alagoas, Maceió, Alagoas, Brazil

*Corresponding author: Erin L. Murphy, emurphy@oceanconservancy.org, 831-440-7958,

Author Contributions: ELM designed research, performed research, contributed tools, analyzed data, and wrote the paper. BRB, NM, GHL, and CMR designed research and wrote the paper. LR designed research, performed research, and wrote the paper. RS performed research and wrote the paper.
Competing Interest Statement: Authors have no competing interests to report
Classifications: Major: Biological Sciences. Minor: Applied Biological Sciences

Keywords: Macroplastic; ingestion; risk assessment

Significance Statement.

Plastic ingestion is a known cause of mortality across taxa, yet the quantitative risk plastic ingestion poses is still poorly understood. Based on data from more than 10,000 necropsies, we estimate the likelihood of mortality due to the gastrointestinal load of various plastic materials—hard, soft, rubber, and fishing debris—for seabirds, marine mammals, and sea turtles. We find that 6 – 405 pieces of ingested macroplastic (or a volume load of between 0.044 – 39.89 cm^3/cm body length), leads to a 90% chance of mortality in these marine species. Importantly, the amount varies depending on plastic types ingested and taxon. Our findings can be used to better understand the mortality risk of macroplastic pollution and inform future risk assessment frameworks.

Supplementary Figures.

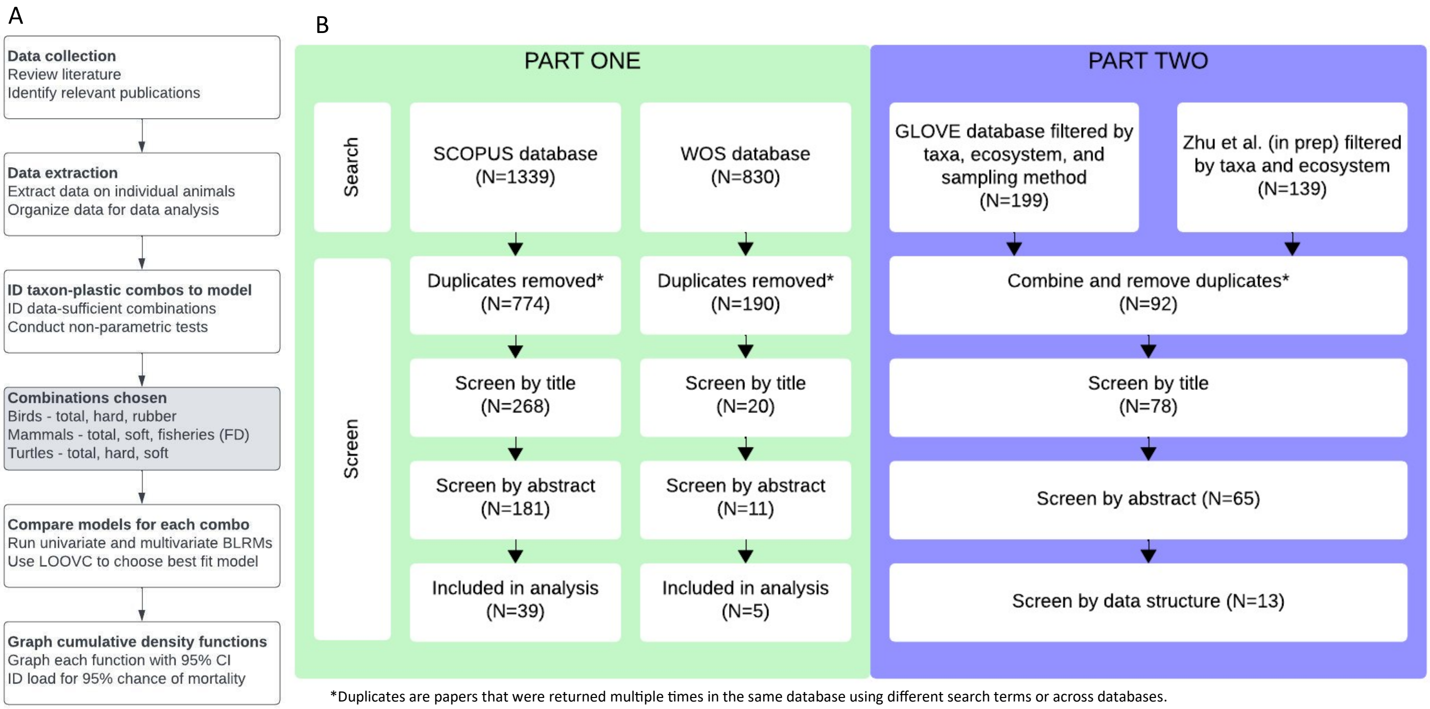

Figure S1. A. Provides an overview of the methods followed in this paper from data collection to model development. The grey box represents the taxa-plastic combinations selected to model. B. The PRISMA flow diagram for our systematic review of the ingestion literature.

Figure S2. Decision tree used for calculating the volume of plastic consumed based on available necropsy data

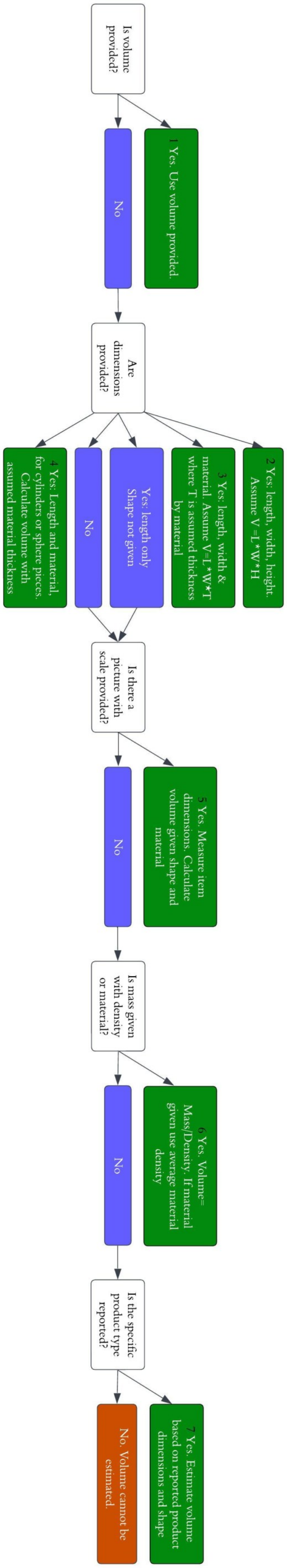

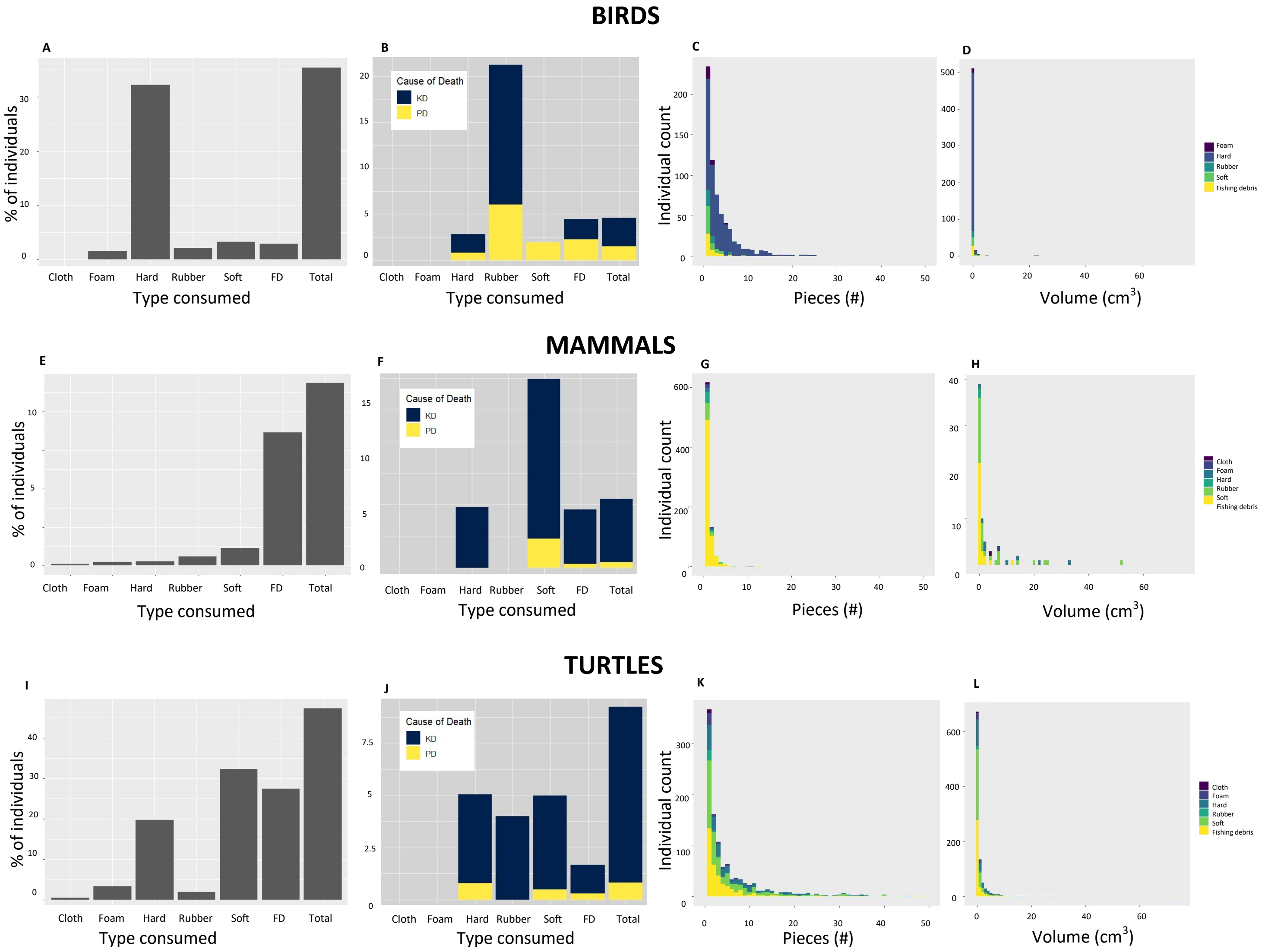

Figure S3. (A), (E), and (I) show the percent of birds, mammals, and turtles, respectively, that ingested plastic by each material type (FD is fishing debris). Of the individuals who consumed a given plastic type, (B), (F), and (J) show the proportion of birds, mammals, and turtles, respectively, that had probable or known debris deaths. (C), (G), and (K) show the number of each plastic type in each individual bird, mammal, and turtle, respectively, for each individual that contained the plastic type, by count. (D), (H), and (L) show the volume of each plastic type in each individual bird, mammal, and turtle, respectively, for individuals that contained a given plastic. Note: x-axis in (H) only goes to 75 cm to allow comparison with birds and turtles, but many mammals ingested greater than 75 cm^3.

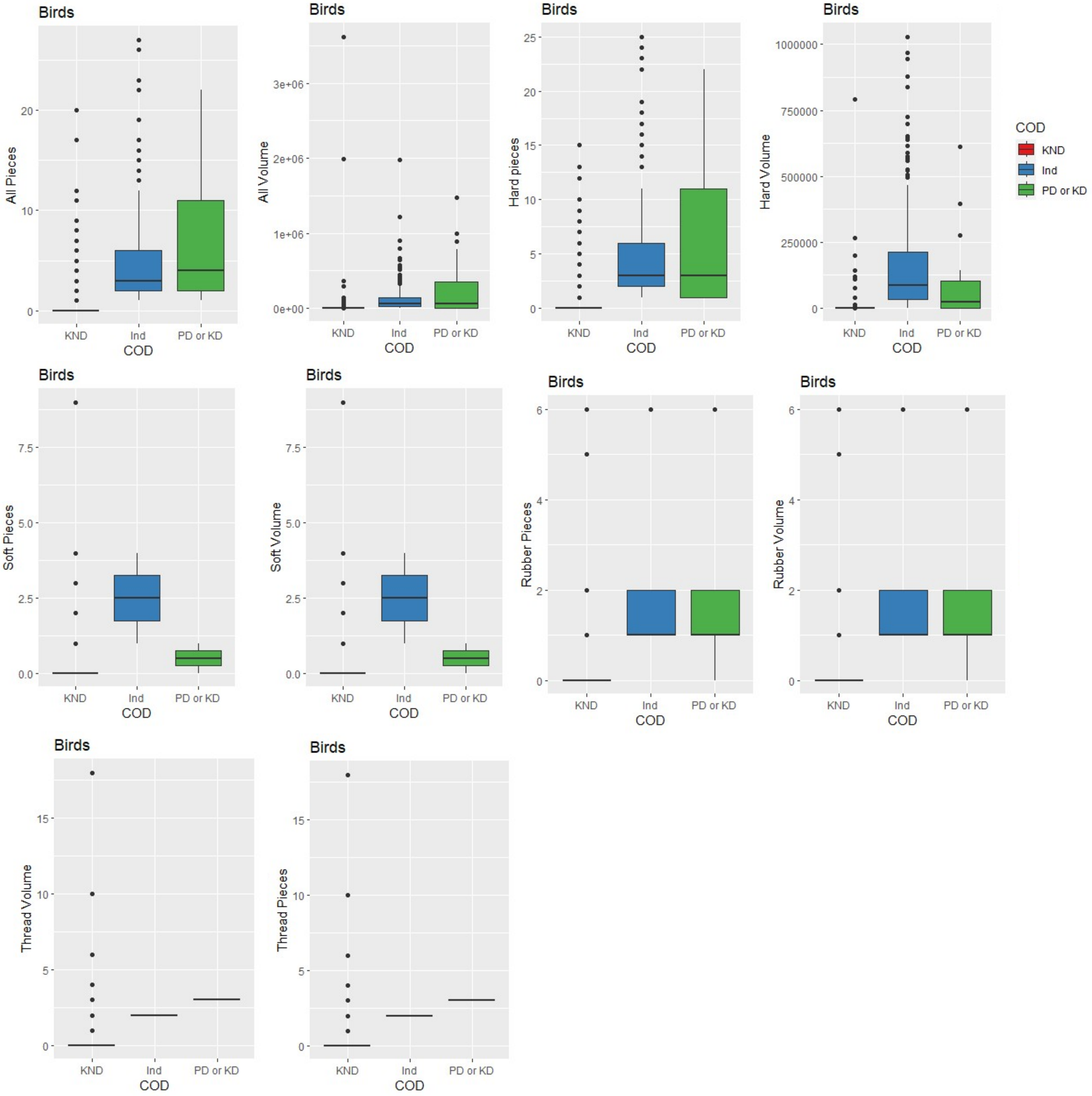

Figure S4. This shows each box plot comparing ingestion amount (pieces and volume) by material type for sea-birds across mortality groups (KND, IND, PD/KD). For all modeled materials, the PD/KD individuals consumed significantly more plastic than the KND individuals.

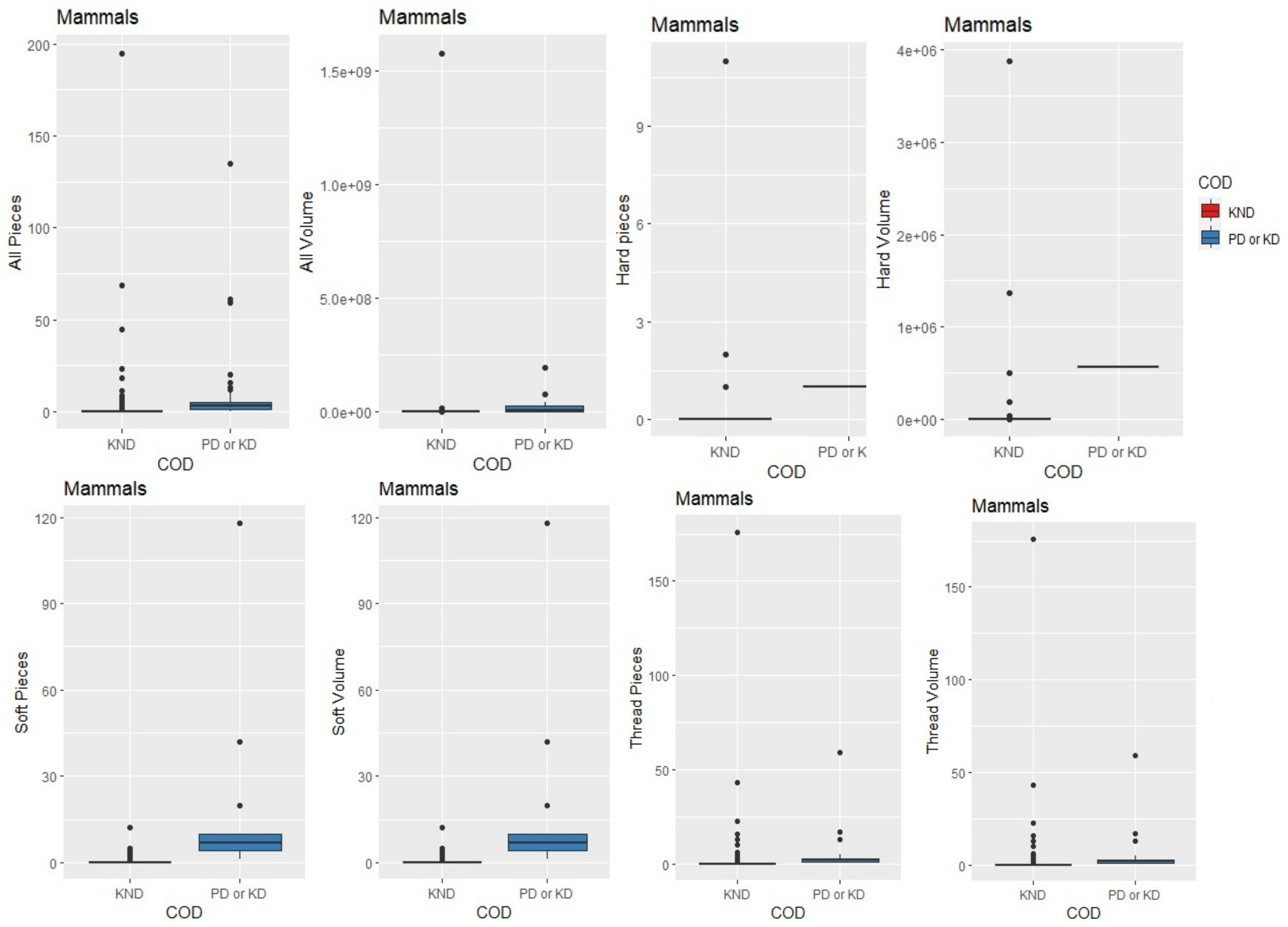

Figure S5. This shows each box plot comparing ingestion amount (pieces and volume) by material type for marine mammals across mortality groups (KND, IND, PD/KD). For all modeled materials, the PD/KD individuals consumed significantly more plastic than the KND individuals.

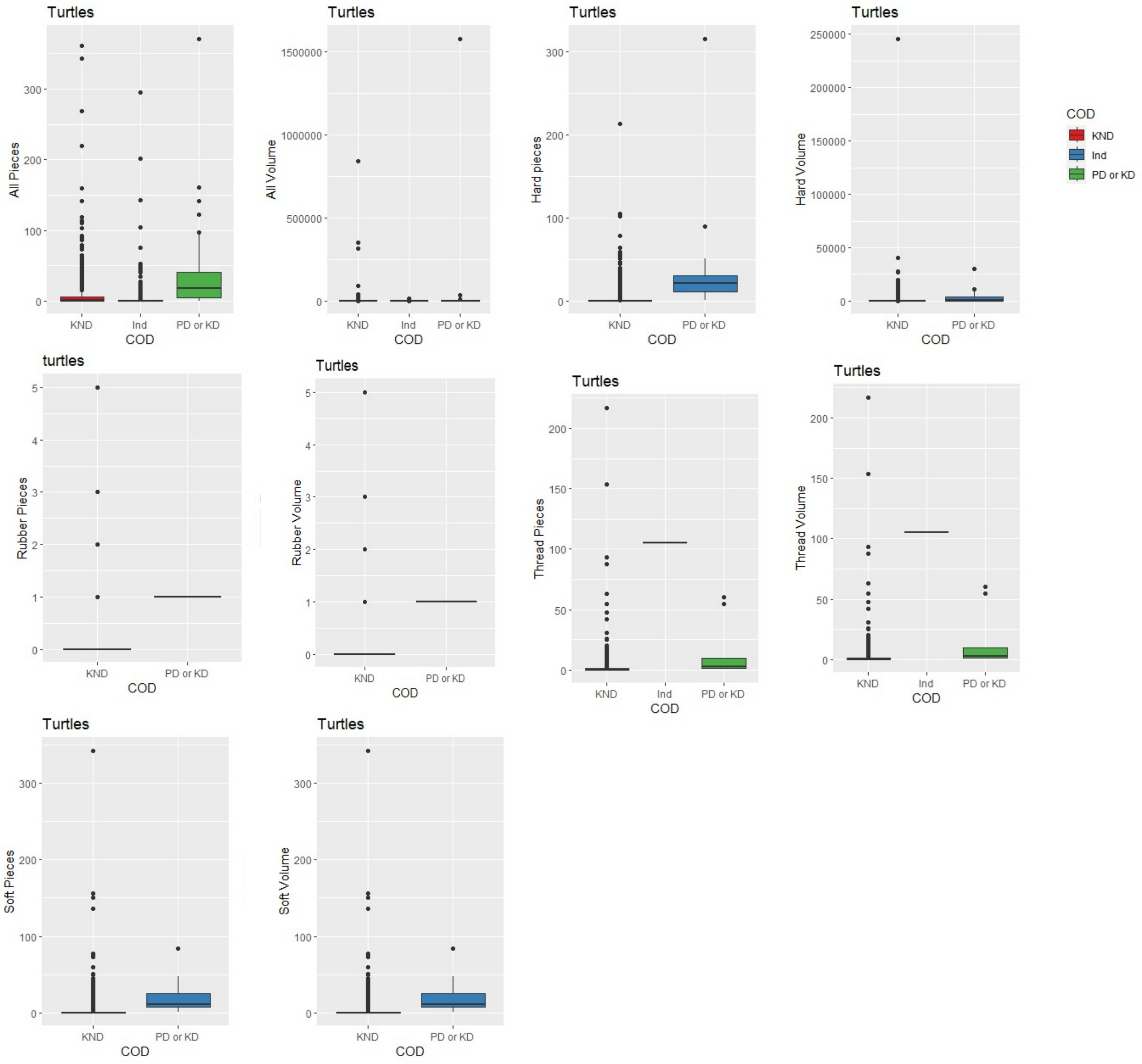

Figure S6. This shows each box plot comparing ingestion amount (pieces and volume) by material type for seaturtles across mortality groups (KND, IND, PD/KD). For all modeled materials, the PD/KD individuals consumed significantly more plastic than the KND individuals.

Table S1. This tables provides the outputs for every model that was produced for each taxon-material-amount type (pieces vs. volume).

**Mortality model**	**#**	β_0	**Log(scale)**	β_1	**50%**	**CI**	**90%**	**CI**	**AIC**
bird pieces	152	2.61	-0.43	NA	10.61	8.44 - 13.47	23.39	17.63 - 28.88	194.27
bird pieces - PD	144	2.71	-0.41	NA	11.65	8.73-16.66	26.1	18.07- 36.59	141.61
bird pieces + IND	544	1.78	-0.24	NA	4.43	4.09 - 4.79	11.35	10.29 - 12.38	2242.46
bird volume/BL cm	131	-3.24	0.094	NA	0.025	0.015 - 0.044	0.098	0.043 - 0.18	-105.75
bird volume/BL cm - PD	123	-2.94	0.097	NA	0.034	0.018- 0.064	0.13	0.050 - 0.25	-60.42
bird volume/BL cm + IND	519	-5.49	0.3	NA	0.0025	0.0022 - 0.0029	0.013	0.0099 - 0.016	-3648.29
bird hard pieces	107	2.66	-0.42	NA	11.3	8.31-15.84	24.76	17.02 - 32.25	113.51
bird hard pieces - PD	103	2.71	-0.42	NA	11.75	8.12 - 20.26	26.01	16.06 - 45.12	86.07
bird hard pieces + IND	486	1.7	-0.22	NA	4.06	3.74 - 4.43	10.64	9.71 - 11.77	2051.71
bird hard volume/BL cm	96	-3.27	0.14	NA	0.019	0.0083 - 0.052	0.095	0.018 - 0.21	-58
bird hard volume/BL cm - PD	93	-3.04	0.11	NA	0.026	0.011 - 0.069	0.12	0.024 - 0.25	-38.71
bird hard volume/BL cm + Ind	436	-5.72	0.22	NA	0.0022	0.0019 - 0.0024	0.009	0.0068 - 0.012	-3289.08
bird rubber pieces	17	1.302	-0.61	NA	2.85	1.81 - 4.47	5.71	2.51 - 6.88	34.96
bird rubber pieces - PD	15	1.44	-0.68	NA	3.33	1.92 - 5.32	6.25	2.56 - 7.71	27.67
bird rubber pieces + IND	31	1.1	-0.42	NA	2.33	1.72 - 3.13	5.17	3.51 - 6.41	86.03
bird rubber vol/BL cm	13	-3.56	-0.57	NA	0.025	0.012 - 0.037	0.043	0.025 - 0.073	-23.24
bird rubber vol/BL cm - PD	12	-3.5	-0.47	NA	0.027	0.011 - 0.047	0.051	0.027 - 0.091	-16.71
bird rubber vol/BL cm + Ind	21	-4.3	0.26	NA	0.0084	0.0036 - 0.018	0.039	0.017- 0.068	-81.9
mammal pieces	898	3.7	-0.072	NA	26.9	15.94 - 47.27	89.25	38.66 - 161.93	456.66
mammal pieces species size	895	2.68	-0.451	6.65E-05	12.3	9-21.4	28.5	16.51 - 58.72	426.03
mammal pieces individual size	897	2.67	-0.289	0.00151	15.15	9.12 - 27.61	42.1	17.11 - 78.84	443.57
mammal pieces species size - PD	891	2.74	-0.452	-6.45E-05	13.06	9.59 - 23.49	30.78	17.67 - 62.08	397.26
mammal vol/length	76	2.23	0.59	NA	4.47	1.84 - 10.98	39.89	15.09 - 80.21	90.16
mammal vol/length - PD	73	2.53	0.384	NA	7.49	3 - 16.25	41.85	17.22 - 76.73	85.69
mammals soft pieces	85	3.36	-0.048	NA	16.35	7.91 - 37.75	62.12	14.82 - 123.37	109.04
mammals soft pieces species size	83	2.46	-0.431	6.42E-05	12.21	7.08 - 22.24	28.52	10.57 - 47.62	94.51
mammals soft pieces individual size	85	2.5	-0.15	0.00184	13.12	6 - 30.94	46.62	8.93 - 87.20	107.95
mammals soft pieces species size - PD	81	2.57	-0.462	6.05E-05	13.03	5.72 - 173.25	32.29	7.89 - 270.44	81.52
mammals soft vol/ body length (cm)	40	0.325	0.378	NA	0.77	0.29-1.93	4.51	1.12 - 7.76	32.13
mammals soft vol/ body length (cm) - PD	38	0.595	0.209	NA	1.16	0.40 - 2.95	5.17	1.2 - 7.8	32.88
mammals thread pieces	645	3.73	-0.011	NA	24.33	11.60 - 57.36	95.69	26.75 - 223.87	289
mammals thread pieces species size	645	2.64	-0.368	6.59E-05	10.28	6.18 - 29.20	28.69	10.65 - 91.37	278.03
mammals thread pieces size	644	2.2	-0.38	0.0018	10.03	5.85 - 21.77	27.82	10.02 - 62.59	275.81
mammal thread pieces size - PD	642	2.22	-0.4	0.0018	10.26	5.85 - 22.95	28.3	9.61 - 64.80	260.96
mammals line length/body length (cm)	35	1.51	-0.22	NA	2.93	0.84 - 7.40	8.73	1.5 - 13.16	19.38
mammals line length/body length (cm) - PD	34	1.74	-0.58	NA	4.26	1.54 - 10.67	9.26	2.29 - 24.07	14.4

Table S2. This tables provides the outputs for every model that was produced for each taxon-material-amount type (pieces vs. volume).

Model	#	β_0	Log(scale)	β_1	50%	CI	90%	CI	AIC
turtle pieces	528	5.13	0.079	NA	113.95	83.33 - 169.39	433.62	266.23 - 730.45	689.98
turtle pieces species size	528	6.21	0.044	-0.0071	118.18	84.28 - 180.41	433.71	258.84 - 742.53	674.07
turtle pieces size	523	3.6	-0.0064	0.042	118.18	83.96 - 179.68	404.5	239.96 - 639.38	656.24
turtle pieces size - PD	519	3.72	-0.014	0.04	125.2	249.58 - 702.06	426.67	249.58 - 702.06	617.66
turtle pieces size + Ind	613	5.55	0.4	-0.023	60.12	46.11 - 80.96	367.07	250.43 - 548.8	1441.11
turtle vol/CCL (cm)	408	0.46	0.36	NA	0.91	0.52 - 1.93	5.52	2.21 - 14	56.5
turtle vol/CCL (cm) - PD	405	0.54	0.34	NA	0.99	0.53 - 2.25	5.85	2.35 - 16.63	59.18
turtle vol/CCL (cm) + Ind	481	0.61	1.15	NA	0.58	0.32 - 1.19	26.43	9.84 -	-424.34
turtle hard/CCL (cm)	170	-0.013	0.11	NA	0.66	0.36 - 1.84	2.88	1.18 - 12.84	24.62
turtle hard/CCL (cm) - PD	168	0.00957	0.022	NA	0.69	0.39 - 1.87	2.72	1.14 -8.22	25.37
turtle hard/CCL (cm) + Ind	180	-0.428	0.2	NA	0.41	0.25 - 0.83	1.93	0.90 - 4.72	15.98
turtle soft pieces	345	4.98	-0.021	NA	96.95	53.90 - 219.73	357.62	138.95 - 853.32	256.56
turtle soft pieces species size	345	5.34	-0.025	-0.0025	98.96	54.16 - 227.21	362.84	138.23 - 862.43	258.29
turtle soft pieces size	342	4.77	-0.053	0.00487	93.37	46.13 - 214.12	341.9	104.56 - 765.37	93.37
turtle soft pieces size - PD	341	4.72	0.12	0.0073	94.99	44.68 - 234.00	347.32	92.16 - 895.85	237.48
turtle soft pieces size + Ind	394	5.37	0.23	-0.27	45.18	30.5 - 71.67	210.08	115.65 - 372.52	682.29
turtles young pieces	282	4.83	0.00095	NA	84.97	57.50 - 133.98	294.36	161.17 - 531.91	353.67
turtles young pieces + species size	282	7.35	-0.022	-0.014	105.2	62.66 - 242.62	377.49	143.01 - 998.16	322.69
turtles young pieces + size	282	2.69	-0.12	0.08	84.83	56.05 - 164.45	266.67	130.29 - 560.51	334.08
turtles young pieces + species size - PD	280	7.31	-0.013	-0.014	108.56	387.24	387.24	147.39 - 979.43	308.92
Young turtle pieces + species size + Ind	296	6.82	0.087	-0.013	76.91	51.48 - 129.51	291.3	142.51 - 577.62	9.29

Table S3. Guidance on the column names and data in the supporting CSV

Column name	Unit	Notes
Age	NA	Age class if provided in manuscript, or if individual size was provided age class was input based on species life history data
Size	Cm	CCL for turtle, total length for mammals, head length for birds
Size_avg	Kg	Average species weight for adult
Size_age	Cm	CCL for turtle, total length for mammals, head length for birds, when data was not available in the manuscript, average size for a species based on age class was used
Length	Cm	Body length for seabirds and marine mammals, CCL for birds. Individual length is used when available, followed by average species length by age class.
COD	NA	Cause of death; KND = known not debris, PD = probably debris, KD = Known debris
Item_gen and item_spec	NA	These represent the items that led to mortality when relevant. Gen = general category (e.g., soft, hard), spec = specific item (e.g., fishing line, plastic bag)
Harddeath, softdeath, etc.	NA	Follows COD categories based on item type cause death. Adds KDO and PDO for columns where an animal died of the material, but a specific material was not cause of death (e.g, film causes death then softdeath is KD and harddeath is KDO)
Hard, soft, etc.	#	Number of pieces found based on category. Note:hard_wo_nurdle + nurdle = hard, rubber_nb + balloon = rubber,. Thread includes line, rope, net, and packing straps
Hard_volume, soft_volume, etc.	cm^3	Volume by material type. Line_length is only dimension given in length and not volume
Id	NA	Author ID for individual from paper
Notes	NA	Information on material consumed
vol_calculation_see_SM fig1	#	Associated with SM Fig 1. shows the method used to calculate volume

Table S4. Frequency of occurrence of plastic in all species (n=95) within our dataset. Values are given as a proportion and percent. *Note there are less individuals here than in the complete dataset, as some individuals were only reported at the genus level.

**Seabird species**		Manx Shearwater	25% (5/20)	Fin Whale	66% (2/3)
American Oystercatcher	100% (1/1)	Neotropic Cormorant	0% (0/3)	Florida Manatee	16.0% (790/4950)
Antarctic Prion	82.4% (14/17)	New Zealand White-capped Albatross	0% (0/82)	Fraser's Dolphin	100% (1/1)
Antipodes Albatross	0% (0/0)	Northern Fulmar	55.2% (16/29)	Gervais' Beaked Whale	100% (2/2)
Arctic Glaucous Gull	9.1% (2/22)	Northern Gannet	100% (1/1)	Gray Seal	0% (0/119)
Atlantic Yellow-nosed Albatross	50% (3/6)	Northern Royal Albatross	0% (0/27)	Guiana Dolphin	2.6% (2/78)
Black Guillemot	0% (0/30)	Red-billed Tropicbird	0% (0/1)	Harbor Porpoise	1.8% (10/554)
Black-browed Albatross	12.5% (1/8)	Red-fronted Coot	0% (0/2)	Harbor Seal	0.64% (6/942)
Black-crowned Night Heron	0% (0/2)	Ruddy Turnstone	0% (0/1)	Harp Seal	0% (0/10)
Black-legged Kittiwake	15% (3/20)	Salvin's Albatross	0% (0/35)	Hooded Seal	12.5% (1/8)
Black-necked Swan	0% (0/1)	Salvin's Prion	75% (9/12)	La Plata Dolphin	15.7% (14/89)
Broad-billed Prion	23.1% (3/13)	Short-tailed Albatross	66% (4/6)	Northern Bottlenose Whale	20% (2/10)
Brown Booby	15.4% (2/13)	Short-tailed Shearwater	85.3% (295/346)	Orca	100% (1/1)
Brown Noddy	0% (0/5)	Shy Albatross	5.9% (2/34)	Pygmy Sperm Whale	5.7% (2/35)
Brown Skua	0% (0/4)	Slender-billed Prion	61.8% (42/68)	Risso's Dolphin	14.3% (4/28)
Buller's Albatross	1.1% (1/90)	Sooty Albatross	0% (0/2)	Rough-toothed Dolphin	13.6% (3/22)
Cabot's Tern	11.1% (3/27)	South American Tern	0% (0/3)	Short-finned Pilot Whale	0% (0/42)
Chatham Island Albatross	0% (0/17)	South Georgia Diving Petrel	0% (0/10)	South American Fur Seal	6.8% (9/133)
Common Tern	0% (0/9)	Southern Giant Petrel	0% (0/1)	Southern Right Whale	100% (1/1)
Cory's Shearwater	80% (4/5)	Southern Royal albatross	25% (1/4)	Sowerby's Beaked Whale	100% (1/1)
Fairy Prion	28.8% (84/292)	Thick-billed Murre	0% (0/30)	Sperm Whale	31.8% (21/66)
Fluttering shearwater	9.4% (8/85)	Wandering Albatross	0% (0/11)	Stejneger's Beaked Whale	5.9% (1/17)
Great Crested Grebe	20% (1/5)	Wedge-tailed Shearwaters	54% (13/24)	Striped Dolphin	3.2% (6/187)
Great Egret	50% (2/4)	White-capped Albatross	0% (0/6)	Tropical Bottlenose Whale	75% (3/4)
Great Shearwater	100% (1/1)	White-chinned Petrel	25% (1/4)	**Turtle species**	
Grey-headed Albatross	30% (3/10)	**Mammal species**		Flatback	46.4% (13/28)
Hutton's Shearwater	0% (0/4)	Atlantic Spotted Dolphin	1.2% (1/82)	Green	45.4% (503/1107)
Kelp Gull	0% (0/2)	Blainville's Beaked Whale	100% (1/1)	Hawksbill	25% (7/28)
Least Grebe	0% (0/2)	Bottlenose Dolphin	8.3% (5/60)	Kemp's Ridley	100% (5/5)
Light-mantled Sooty Albatross	17.6% (3/17)	Common Dolphin	10.9% (6/55)	Leatherback	5.7% (4/7)
Little Shearwater	50% (4/8)	Common Minke Whale	100% (1/1)	Loggerhead	67.2% (39/58)
Magellanic Penguin	23.3% (10/43)	Cuvier's Beaked Whale	2.9% (1/34)	Olive Ridley	6.4% (47/73)
Magnificent Frigatebird	0% (0/4)	Deraniyagala's Beaked Whale	100% (1/1)		

Table S5. This table provides the p-value and Adjusted R^2 values for total mass vs. total volume found in each individual included in the model by taxa. The two were highly correlated across taxa; however rows were excluded given that many individuals did not have mass data available.

Taxa	P-value	Adj. R^2
Seabird	< 2.2e-16	0.47
Marine mammal	< 2.2e-16	0.91
Sea turtle	< 2.2e-16	0.99

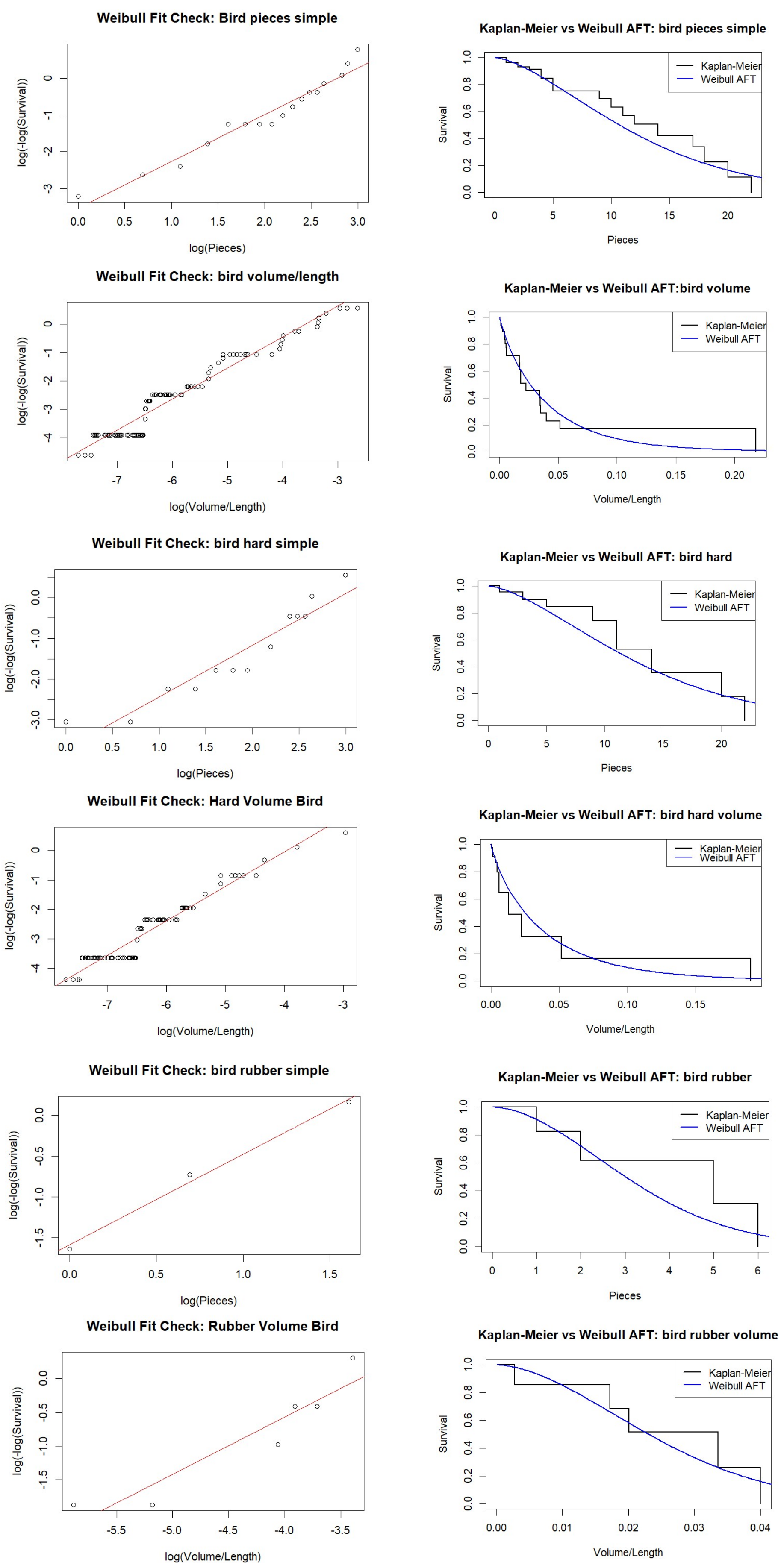

Figure S7. Supplemental figures showing the fit of the Weibull distribution on the bird data for each model as well as the comparison between the Kaplan-Meier non-parametric model and the Weibull AFT parametric model.

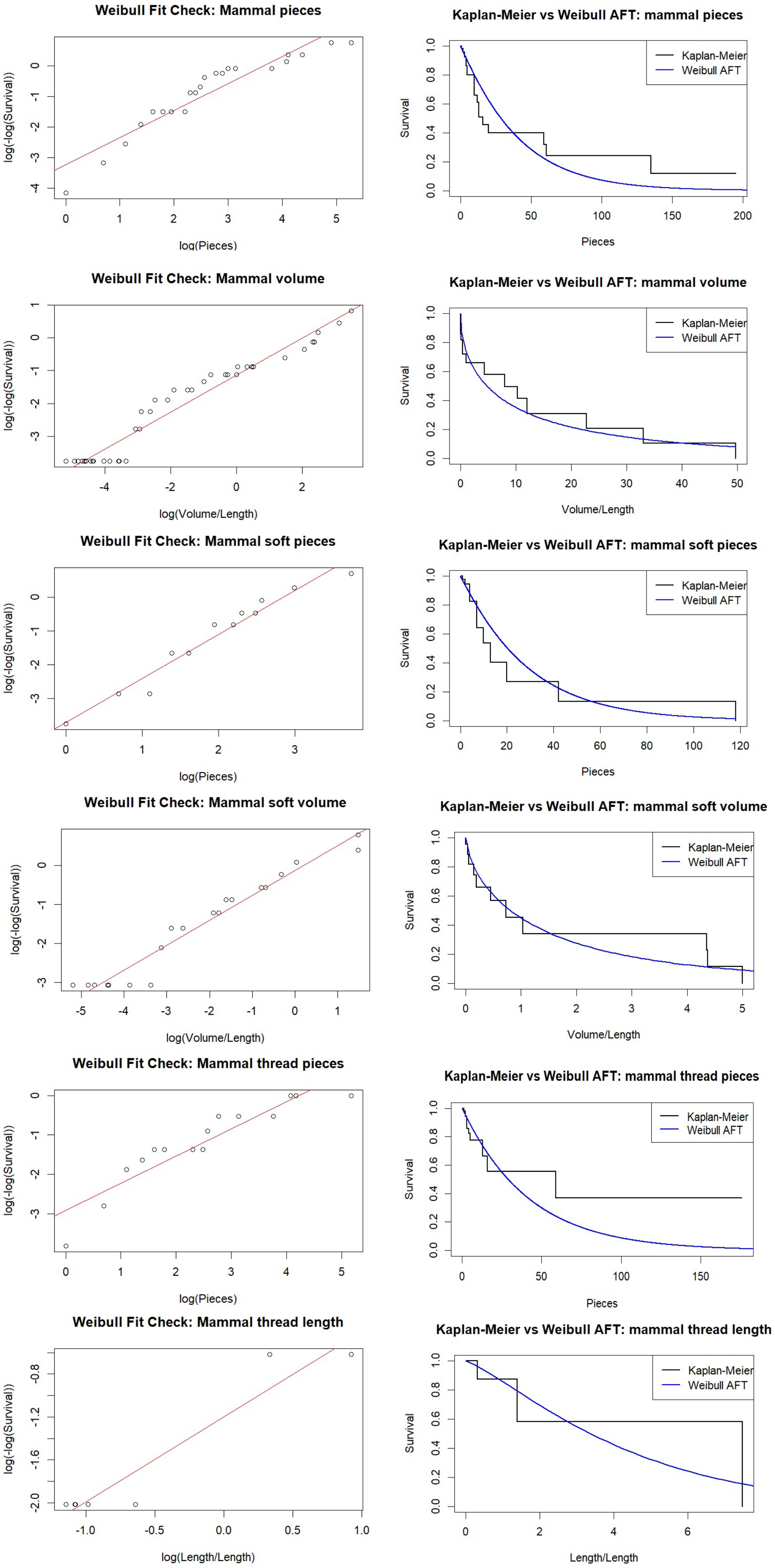

Figure S8. Supplemental figures showing the fit of the Weibull distribution on the mammal data for each model as well as the comparison between the Kaplan-Meier non-parametric model and the Weibull AFT parametric model.

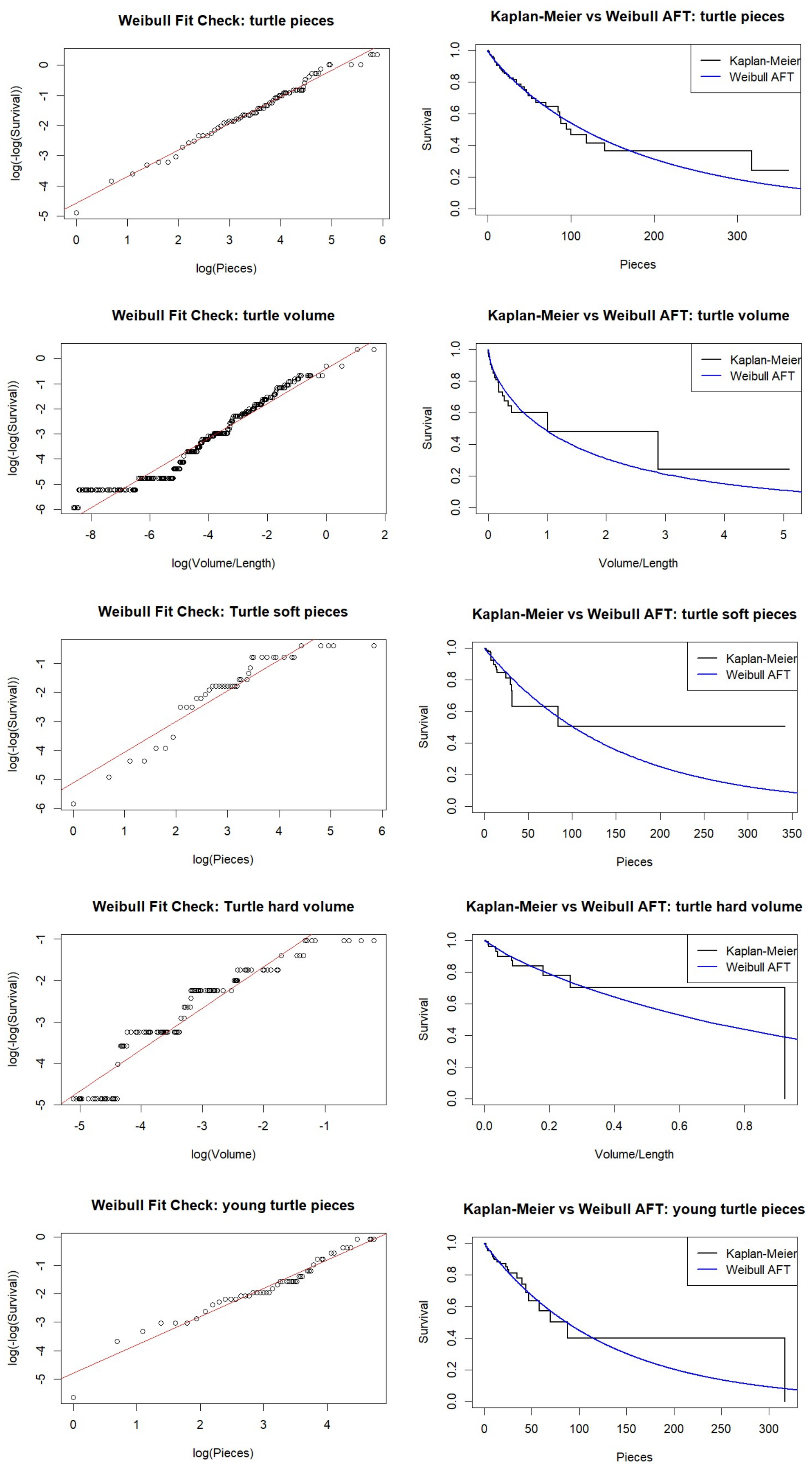

Figure S9. Supplemental figures showing the fit of the Weibull distribution on the turtle data for each model as well as the comparison between the Kaplan-Meier non-parametric model and the Weibull AFT parametric model.
